# Reduced microglia activation following metformin administration or microglia ablation is sufficient to prevent functional deficits in a mouse model of neonatal stroke

**DOI:** 10.1186/s12974-022-02487-x

**Published:** 2022-06-15

**Authors:** Clara Bourget, Kelsey V. Adams, Cindi M. Morshead

**Affiliations:** 1 Institute of Medical Sciences, University of Toronto, Toronto, M5S1A8 Canada; 2 Donnelly Centre for Cellular and Biomolecular Research, University of Toronto, 160 College Street, Room 1006, Toronto, ON M5S3E1 Canada; 3 Division of Anatomy, Department of Surgery, University of Toronto, Toronto, M5S1A8 Canada; 4 Institute of Biomedical Engineering, University of Toronto, Toronto, M5S3E1 Canada

**Keywords:** Neonatal Stroke, Mouse, Microglia, Astrocytes, Metformin, Plexxikon 5622, Motor Deficits

## Abstract

**Background:**

Neonatal stroke is a devastating insult that can lead to life-long impairments. In response to hypoxic–ischaemic injury, there is loss of neurons and glia as well as a neuroinflammatory response mediated by resident immune cells, including microglia and astrocytes, which can exacerbate damage. Administration of the antidiabetic drug metformin has been shown to improve functional outcomes in preclinical models of brain injury and the cellular basis for metformin-mediated recovery is unknown. Given metformin’s demonstrated anti-inflammatory properties, we investigated its role in regulating the microglia activation and used a microglia ablation strategy to investigate the microglia-mediated outcomes in a mouse model of neonatal stroke.

**Methods:**

Hypoxia-ischaemia (H-I) was performed on post-natal day 8. Metformin was administered for one week, starting one day after injury. Immunohistochemistry was used to examine the spatiotemporal response of microglia and astrocytes after hypoxia-ischaemia, with or without metformin treatment. To evaluate the effects of microglia depletion after hypoxia-ischaemia, we delivered Plexxikon 5622 for 1 or 2 weeks post-injury. The regional pattern of microglia and astrocyte depletion was assessed through immunohistochemistry. Motor behaviour was assessed with the righting reflex, hindlimb suspension, grip strength and cylinder tests.

**Results:**

Herein, we revealed a spatiotemporally regulated response of microglia and astrocytes after hypoxia-ischaemia. Metformin treatment after hypoxia-ischaemia had no effect on microglia number and proliferation, but significantly reduced microglia activation in all regions examined, concomitant with improved behavioural outcomes in injured mice. Plexxikon 5622 treatment successfully ablated microglia, resulting in a > 90% depletion in microglia in the neonatal brain. Microglia rapidly repopulated upon treatment cessation of Plexxikon. Most interesting, microglia ablation was sufficient to reduce functional deficits after hypoxia-ischaemia, mimicking the effects of 1 week of metformin treatment post-injury.

**Conclusion:**

These results highlight the importance of regulating the neuroinflammatory response after neonatal stroke to promote recovery.

**Supplementary Information:**

The online version contains supplementary material available at 10.1186/s12974-022-02487-x.

## Introduction

Neonatal stroke is an important cause of brain injury that occurs in ~ 1 in 2300 infant births [12, 17, 45]. Children who suffer from this form of hypoxic–ischaemic (H-I) brain injury exhibit long-term neurologic complications ranging from mild behavioural deficits to severe seizures, developmental delays and/or cerebral palsy [19, 20, 40, 59]. Neural injury results from excitotoxicity, oxidative stress and inflammation, leading to a loss of oligodendrocytes and neurons, both of which are particularly sensitive to hypoxic–ischaemic insult [1–4, 10, 29, 37]. Neonatal stroke, despite its prevalence and significant impact on the health care system, is less well studied than adult stroke. The current treatment for neonatal stroke is hypothermia; however, the window of efficacy severely limits its potential to reduce the damage that results from the insult [41, 51]. The need for novel interventions to limit the damage and promote neural repair is clear.

Metformin, a commonly used drug to treat type-2 diabetes, has been shown to have pleiotropic effects in the brain. Our group, and others, have shown that metformin administration leads to expansion of the endogenous neural precursor pool in their subependymal zone (SEZ) niche, migration of neural precursor cells into stroke-injured parenchyma, contribution to neurogenesis and oligodendrogenesis and improved motor and cognitive outcomes [10, 48]. Metformin has also been shown to promote neurogenesis and reduce neuroinflammation in a model of juvenile cranial radiation [11, 38], similar to other post-natal and adult models of brain injury where metformin’s anti-inflammatory properties have been demonstrated by regulating the AMPK-NF-Kb-dependent pathway [23, 57]. The promising effects of metformin to support brain repair were further demonstrated in a study that examined metformin-mediated recovery in a model of cranial radiation and a pilot clinical trial that demonstrated the safety and feasibility of metformin for children with acquired brain injury [3]. Given the pleiotropic effects of metformin, the cellular basis for the improved outcomes following perinatal brain injury and metformin administration is unclear.

Microglia are the resident immune cells of the central nervous system and during brain maturation, microglia perform several functions, such as synaptic pruning and supporting axon myelination [22, 43, 54, 60]. In the uninjured post-natal brain, microglia adopt a ramified morphology with dynamic process movements, allowing them to sense the microenvironment [39]. It has been shown in the uninjured adult brain that microglia exhibit regional differences in terms of cell density, as well as inflammatory profiles. For example, cortical and striatal microglia display higher degrees of ramification, are less proliferative and less phagocytic than SEZ microglia [34, 46, 55, 56, 58]. Following injury, microglia become activated, a process that includes proliferation and pronounced de-ramification to amoeboid shaped cells, as well as increased cytokine release and phagocytosis, all of which are processes that can exacerbate brain damage [18, 31, 42, 63].

The neuroinflammatory response after injury has also been shown to be dependent on astrocytes. Similar to microglia, astrocytes have pro-inflammatory and immunoregulatory (neuroprotective) subpopulations [52]. Astrocyte activation results in their hypertrophy, upregulation of GFAP and in severe insult, can result in their proliferation and the formation of a glial scar. Once activated, they can exacerbate injury by inhibiting neural progenitor homeostasis, impairing synapse homeostasis and increasing cytokine release [50, 64]. Notably, astrocytes activation has been shown to be dependent on microglia activation both in vivo and in vitro [24, 27, 30].

In this study, we aimed to shed light on metformin's cellular targets following neonatal stroke. We characterized the microglia and astrocyte response, with and without metformin treatment, using a well-established model of neonatal stroke. Herein, we show that the microglia response is regionally distinct at early times post-stroke and metformin treatment reduces microglia activation, as measured by morphology and protein expression (CD68). Metformin treatment after neonatal stroke did not affect astrocyte activation. Using a novel model of microglia ablation, we show that the loss of microglia does not result in motor behaviour impairments in early post-natal mice. Most interesting, the loss of microglia protects against the functional deficits that result following neonatal stroke, mimicking the metformin-mediated outcomes after 1 week of metformin treatment post-stroke. Our work provides novel insight into metformin's anti-inflammatory effects after neonatal stroke and further highlights the role of microglia in the observed motor impairments following injury to the early post-natal brain.

## Methods

### Animals

Transgenic GFAP::GFP mice that express green fluorescent protein under the control of the human astrocyte GFAP promoter were used for all experiments (Zhuo et al. 1997). The mice were housed on a 12-h (h) day/night light cycle with access to food and water ad libitum, and each cage contained a dome house. Mice were bred in-house, and each dam was provided with a nestlet. A total of 141 neonatal, male and female mice were used. All animal procedures were in accordance with institutional guidelines and approved by the University of Toronto Animal Care Committee, following the guidelines of the Canadian Council for Animal Care.

### Surgical procedure

Post-natal day-8 (P8) mice were anaesthetized with isoflurane (5% induction and 1.5% maintenance) and the hypoxia–ischaemia (H-I) injury was performed as previously described [10, 33, 48]. Briefly, the left common artery was ligated using 6–0 sutures. Pups were administered bupivacaine (0.25 mg/ml) during the operation to provide local anaesthesia. Following the surgery, mice were placed under a heat lamp during recovery and were then returned to their home cages for 1 h. Subsequently, mice were placed in an 8% hypoxia chamber for 1 h at 37 °C. Mice were returned to their home cages, and 1 h post-H-I they performed the righting reflex to assess motor behaviour.

### Drug administration

#### Metformin

Metformin (1,1-dimethylbiguanide hydrochloride; D150959, Sigma-Aldrich, Oakville, ON, Canada) was administered daily via subcutaneous injection directly to the neonates (200 mg/kg) [10, 11, 33, 48] from P9 to P14. Metformin was dissolved in sterile phosphate-buffered saline (PBS) for a final concentration of 20 mg/ml.

#### EdU

Pups received an intraperitoneal injection of EdU (5-ethynyl-2'-deoxyuridine; E10187, Fisher Scientific, Pittsburgh, PA, USA) (50 mg/kg at a concentration of 10 mg/ml, dissolved in PBS) one hour before sacrifice.

#### Plexxikon 5622

Plexxikon 5622 (S8874, Sellckchem, Houston, TX, USA) (stock solution, 79 mg/ml; dissolved in dimethyl sulfoxide (DMSO) (4540, Sigma-Aldrich) was injected daily via intraperitoneal injections from P8 to P15 at a final concentration of 50 mg/kg in sterile PBS and 20% Kolliphor40 (07,076, Sigma-Aldrich). Control mice received vehicle injections of DMSO with sterile PBS and 20% Kolliphor40.

### Tissue preparation and immunohistochemistry

#### Tissue preparation

Animals were sacrificed on P9, P12, P14, P15, P21 and P22 with an overdose of Avertin. Transcardial perfusions were performed using cold PBS followed by 4% paraformaldehyde (PFA; P6148, Sigma-Aldrich). The brains were removed and post-fixed in 4% PFA overnight, then cryoprotected in 30% sucrose solution and stored at 4 °C until cryosectioning. Brain sections (coronal, 20um) were mounted on Superfrost Plus microscope slides (12-550-15, Fisher Scientific) and kept at − 20 °C until processing.

#### Immunohistochemistry

Slides were thawed and rehydrated in PBS for 5 min at room temperature. Slides were placed in 0.3% Triton X-100 (T9284, Sigma-Aldrich) for 20 min followed by 3 X 5-min PBS washes. For EdU, slides were incubated for 30 min with the Click-It reaction cocktail, which was composed of Tris-buffered saline (6.05 g Tris (17,926, Thermo Fisher Scientific) + 8.76 g NaCl (SOD001.1, BioShop, Canada) + 800 ml ddH2O; pH of 7.5), Copper Sulphate (2411A, Sigma Aldrich), 647 Azide (A10277, Fisher Scientific) and 1X reaction buffer (C10634, Thermo Fisher Scientific). Next, slides were washed with PBS and were incubated in a blocking solution made of included 500ul normal goat serum (NBP2-23,475, Novus Biologicals Canada, Toronto, ON, Canada), 100 mg bovine serum albumin (A9647, Sigma-Aldrich) and 9.5 ml of 0.3% Triton X-100 (T9284, Sigma-Aldrich) for 1 h at room temperature. Following the 1 h block, primary antibodies were prepared in the blocking solution and sections were incubated overnight at 4 °C. Primary antibodies included rabbit-anti-Iba1 (Iba1) (1:500; 019–19,741, Fujifilm WAKO, Richmond, VA, USA), rat-anti-mouse CD68 (CD68) (1:200; MCA1957, Bio Rad, Hercules, CA, USA), chicken anti-GFP (GFP) (1:500; GFP-1020, Aves Lab, Davis, CA, USA) and rabbit anti-GFAP (1:500; G9269, Sigma-Aldrich). The next day, sections were washed 3 X 5 min with 0.2% Tween 20 (P9416, Sigma-Aldrich) in PBS, followed by a 1 h secondary antibody incubation at room temperature. Secondary antibodies included goat anti-rabbit Alexa Fluor 568 (1:500; A11036, Fisher Scientific) for Iba1 and GFAP (used on different slides), goat-anti-rat Alexa Fluor 647 (1:500; A21247, Thermo Fisher Scientific) for CD68 and goat anti-chicken Alexa Fluor 488 (1:500; A11089, Thermo Fisher Scientific) for GFP, dissolved in PBS. Slides were washed 3 × 5 min in PBS and incubated with DAPI (1:10,000; D1306, Thermo Fisher Scientific) dissolved in PBS for 5 min to label nuclei. A final 3 X 5-min wash in PBS was performed and slides were coverslipped with mounting medium (S302380-2, Dako, Santa Clara, CA, USA) and stored at − 20 °C until imaging.

### Neurosphere assay

The in vitro neurosphere assay was performed to assess the numbers of neural stem cells, as previously described [8]. Briefly, the periventricular region was dissected; cells were mechanically dissociated and placed in an enzyme solution containing 1.33 mg/ml trypsin (T1005, Sigma-Aldrich), 0.76 mg/ml hyaluronidase (H6254, Sigma-Aldrich) and 0.13 mg/ml kynurenic acid (K3375, Sigma-Aldrich) in artificial cerebrospinal spinal fluid [8]. Cells were incubated for 25 min at 37 °C, followed by centrifugation for 5 min at 1500 rpm. The supernatant was removed, and samples were placed in a trypsin inhibitor solution consisting of 0.67 mg/ml ovomucoid (LS003086, Worthington, Lakewood, NJ, USA) in serum-free media (SFM) containing l-glutamine (2 mM; Invitrogen, Waltham, MA, USA) and penicillin/streptavidin (100 U/0.1 mg/ml; Invitrogen) [8]. Samples were triturated and centrifuged for 5 min at 1500 rpm, then added to SFM supplemented with epidermal growth factor (20 ng/ml; PMG8041, GIBCO, Pittsburgh, PA, USA), basic fibroblast growth factor (10 ng/ml; PHG0266, GIBCO) and heparin (2ug/ml; H3149, Sigma-Aldrich), plated at 10 cells/ul (Tabake-Coles et al. 2008) and incubated for 7 days at 37 °C and 5% CO_2_. The numbers of neurospheres (> 80um in diameter) were counted.

### Imaging and quantification

Slides were imaged using an Axiovert 200 M inverted microscope (Zeiss, Jena, Germany) and an Axio Observer Z1 inverted motorized microscope (Zeiss, Jena, Germany) with a high-speed spinning disk CSU-X1 confocal scanner unit (Yokogawa, Texas, USA) and an Axiocam 506 high-resolution camera (Zeiss, Jena, Germany). Images were acquired with the AxioVision 4.8 and Zen software (Zeiss, Germany). Three to four sections were counted per brain. Three regions of interest were imaged in the cortex and striatum (each 350 × 300 um; 0.105 mm^2^) and the lateral wall of the lateral ventricles (150 × 300 um; 0.045 mm^2^) (including the dorsolateral corner), in the ipsilateral and contralateral hemispheres (relative to the ischaemic insult). Areas chosen extended from the crossing of the corpus callosum anteriorly to the crossing of the anterior commissure, posteriorly.

Amoeboid microglia were defined as DAPI + cells with a rounded cell body morphology with and a lack of processes (≤ 1 process less than 5um in length) extending from the soma. Cells in the cortex were manually counted using the Cell Counter plugin in Fiji (Schindelin et al., 2012). These counts were expressed as fold changes and percentages of cells per unit area for each of the regions.

To measure microglia intensity and soma size, images were exported as TIFF files and sections were converted to 8 bits and filtered (filter = MaxEntropy). Pixel counts and soma length measurements were generated for Iba1 + cells (1) in the cortex (each 350 × 300 um; 0.105 mm^2^), (2) in the striatum (each 350 × 300 μm; 0.105 mm^2^) and (3) and in the dorsolateral corner of the lateral ventricle (150 × 300 um; 0.045 mm^2^) using the pixel inspection tool and measurement tool in Fiji, respectively, from > 3 sections per brain.

### Behavioural assessments

Mice were handled on the day prior to behavioural testing in all tasks. On the day of testing, mice were given 30 min to acclimatize to the room before testing.

### Righting reflex

The righting reflex was performed 1 h post-H-I [16]. Mice were placed in the supine position and the time needed to turn into prone position was recorded (maximum 60 s) and averaged over 3 trials.

#### Hindlimb suspension

On P12, mice performed the hindlimb suspension test, which assesses hindlimb strength [10, 16]. Mice were placed head-down on the edge of a 50-ml falcon tube, with tissue at the bottom. Mice were suspended on the edge of the tube using their hindlimbs and the latency to fall was recorded for 3 trials (maximum 60 s). The mean latency of the trials was calculated per mouse.

### Grip strength

On P15, mice performed the grip strength test [16]. This test assesses the paw strength of all 4 paws simultaneously. Mice were placed on an 11 × 7 inch grid, and it was rotated from a horizontal to a vertical position. The angle at which the mouse fell from the grid was recorded and averaged over 3 trials.

### Cylinder

On P22, mice performed the cylinder test which assesses functional asymmetry. In this test, mice are placed in a Plexiglas transparent cylinder and recorded for 4 min or until each paw touches the cylinder at least 10 times. Forepaw preference was measured as the number of touches from the left, right or both forepaws. Percent forepaw preference was established using the following equation:

$$\% \;\text{forepaw} \;\text{preference}= \frac{(\text{left}-\text{right})}{(\text{left}+\text{right}+\text{both})}\times100.$$

### Statistical analysis

Statistical analyses were conducted using GraphPad Prism 9 (GraphPad Software, San Diego, CA, USA). All data are graphed as mean ± standard error of the mean (SEM). Statistical analysis consisted of unpaired *t*-tests, one-way ANOVA or two-way ANOVA. Where appropriate, Tukey’s post hoc analysis was used. Statistical significance was established as *p* < 0.05. Experimenters were blinded to the experimental conditions during analyses.

## Results

### Microglia increase in numbers after H-I, with or without metformin, in the cortex and striatum.

**Fig. 1:**
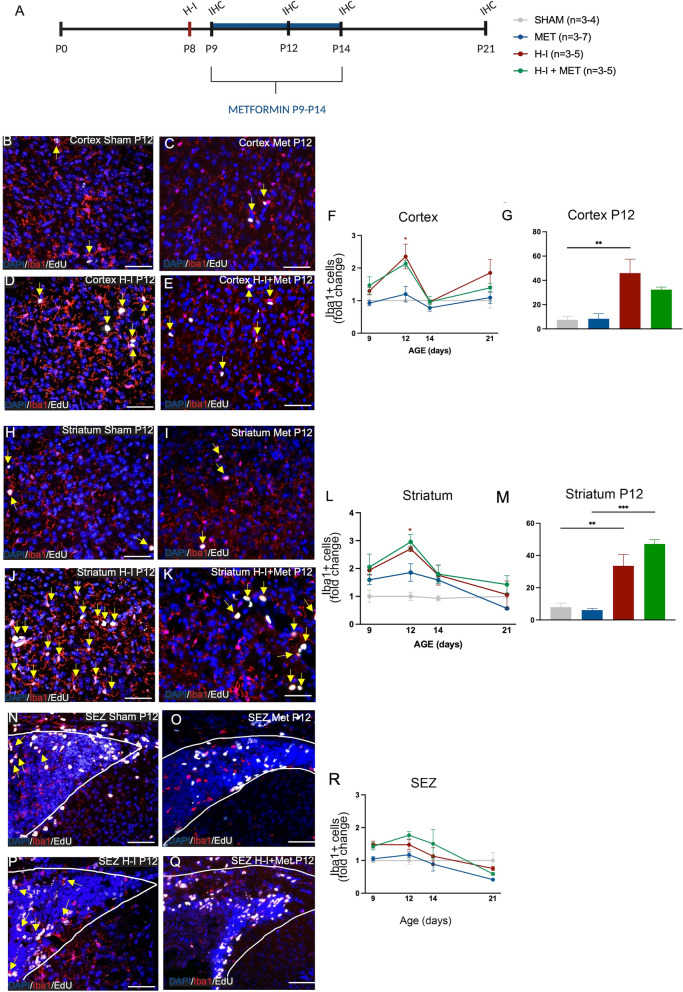
H-I, in the presence or absence of metformin, increases microglia numbers in the cortex and striatum, but not the SEZ. **A** Experimental timeline. Mice received H-I on P8 and metformin administration from P9-P14. Immunohistochemistry (IHC) was performed on P9, P12, P14 and P21.** B** Representative image of microglia (Iba1+, red) and proliferating microglia (Iba1 + (red) EdU + (white) double-positive cells) at P12 in the cortex in Sham mice (×20 magnification). Proliferating microglia are depicted by yellow arrows. **C** Representative image of microglia (Iba1+, red) and proliferating microglia (Iba1 + (red) EdU + (white) double-positive cells) at P12 in the cortex in Met-treated mice (×20 magnification). Proliferating microglia are depicted by yellow arrows. **D** Representative image of microglia (Iba1 +) and proliferating microglia (Iba1 + EdU +) at P12 in the cortex after H-I (×20 magnification). Proliferating microglia are depicted by yellow arrows. Representative image of microglia (Iba1 +) and proliferating microglia (Iba1 + EdU +) at P12 in the cortex after H-I (×20 magnification). Proliferating microglia are depicted by yellow arrows. **E** Representative image of microglia (Iba1 +) and proliferating microglia (Iba1 + EdU +) at P12 in the cortex after H-I + Met treatment (×20 magnification). Proliferating microglia are depicted by yellow arrows. **F** Quantification of the number of microglia (Iba1 + cells) in the cortex across time points and treatment groups expressed as fold change. Microglia were significantly increased after H-I at P12 relative to Sham (1.00 ± 0.05-fold change of Iba1 + cells in Sham mice vs. 2.36 ± 0.38 in H-I-injured mice, p = 0.0063). This trend was observed after HI + Met (1.20 ± 0.24-fold change of Iba1 + cells in Met-treated mice vs. 2.13 ± 0.09-fold change of Iba1 + cells in H-I + Met-treated mice, p = 0.073). Average number of Iba1 + cells/unit area: Sham = 22.69 ± 0.97; Met = 27.88 ± 5.57; H-I = 58.13 ± 4.00, H-I + Met = 50.59 ± 1.77. **G** Quantification of percent proliferating microglia (Iba1 + EdU + /Iba1 +) in the cortex at P12 across treatment groups. A significant increase was observed after H-I (7.50 ± 2.86% Iba1 + EdU + cells/unit area in Sham vs. 45.98 ± 11.45% Iba1 + EdU + cells/unit area in H-I, p = 0.0064) and Met treatment did not prevent this expansion (45.98 ± 11.45% Iba1 + EdU + cells/unit area in H-I vs. 32.38 ± 2.07% Iba1 + EdU + cells/unit area in H-I + Met, p = 0.37). There was no significant difference between Met-treated mice and H-I + Met-treated mice (8.38 ± 4.13% Iba1 + EdU + cells/unit area in Met vs. 32.38 ± 2.07% Iba1 + EdU + cells/unit area in H-I + Met-treated mice, p = 0.056). **H** Representative image of microglia (Iba1 +) and proliferating microglia (Iba1 + EdU +) at P12 in the striatum in Sham mice (×20 magnification). Proliferating microglia are depicted by yellow arrows. **I** Representative image of microglia (Iba1+, red) and proliferating microglia (Iba1 + (red) EdU + (white) double-positive cells) at P12 in the striatum in Met-treated mice (×20 magnification). Proliferating microglia are depicted by yellow arrows. **J** Representative image of microglia (Iba1 +) and proliferating microglia (Iba1 + EdU +) at P12 in the striatum after H-I (×20 magnification). Proliferating microglia are depicted by yellow arrows. **K** Representative image of microglia (Iba1 +) and proliferating microglia (Iba1 + EdU +) at P12 in the striatum after H-I + Met treatment (×20 magnification). Proliferating microglia are depicted by yellow arrows. **L** Quantification of the number of microglia (Iba1 +) in the striatum across time points and groups expressed as fold change. Microglia were significantly increased after H-I at P12 relative to Sham (1.00 ± 0.15-fold change of Iba1 + cells in Sham mice vs. 2.70 ± 0.10 in H-I-injured mice, *p* = 0.032). There was no significant difference in Iba1 + cells in Met-treated mice compared to H-I + Met (1.86 ± 0.31-fold change of Iba1 + cells in Met-treated mice vs. 2.95 ± 0.27-fold change of Iba1 + cells in H-I + Met-treated mice, p = 0.29). Average number of Iba1 + cells/unit area: Sham = 8.25 ± 1.11; Met = 14.97 ± 2.71; H-I = 58.13 ± 4.00, H-I + Met = 27.83 ± 2.13. **M** Quantification of proliferating microglia (Iba1 + EdU + /Iba1 +) in the striatum at P12 across groups. Significant increases were observed between Sham and H-I (7.99 ± 2.41% Iba1 + EdU + cells/unit area in Sham vs. 33.58 ± 7.02% Iba1 + EdU + cells/unit area in H-I, *p* = 0.0085) and Met-treated mice vs. H-I + Met-treated mice (6.12 ± 1.16% Iba1 + EdU + cells/unit area in Met vs. 47.19 ± 2.64% Iba1 + EdU + cells/unit area in H-I + Met, *p* = 0.15). Quantification of proliferating microglia (Iba1 + EdU + /Iba1 +) in the striatum at P12 across groups. Significant increases were observed between Sham (7.99 ± 2.41% Iba1 + EdU + cells/unit area) vs. H-I (33.58 ± 7.02% Iba1 + EdU + cells/unit area) (*p* = 0.009) and Sham (7.99 ± 2.41% Iba1 + EdU + cells/unit area) vs. H-I + Met (47.19 ± 2.64% Iba1 + EdU + cells/unit area) (*p* = 0.006). **N** Representative image of microglia (Iba1+) and proliferating microglia (Iba1 + EdU +) at P12 in the SEZ in Sham mice (×20 magnification). Proliferating microglia are depicted by yellow arrows. **O** Representative image of microglia (Iba1+, red) and proliferating microglia (Iba1 + (red) EdU + (white) double-positive cells) at P12 in the striatum in Met-treated mice (×20 magnification). Proliferating microglia are depicted by yellow arrows. **P** Representative image of microglia (Iba1 +) and proliferating microglia (Iba1 + EdU +) at P12 in the SEZ after H-I (×20 magnification). Proliferating microglia are depicted by yellow arrows. **Q** Representative image of microglia (Iba1 +) and proliferating microglia (Iba1 + EdU +) at P12 in the SEZ after H-I + Met treatment (×20 magnification). Proliferating microglia are depicted by yellow arrows. **R** Quantification of the number of microglia (Iba1 +) in the SEZ across time points and groups expressed as fold change. No significant differences were found between groups (*p* = 0.15, *n* = 3–7) mice per group. Data presented as mean ± SEM. Scale Bar: 50um. Unit area (cortex, striatum): 3 × 0.105 mm^2^. Unit area (SEZ): 3 × 0.045mm2. Statistics: (D, H, L) Two-way ANOVA; (E, I) One-way ANOVA. *p ≤ 0.050

Our group has previously demonstrated that one week of metformin treatment is sufficient to reduce motor functional deficits following neonatal stroke [10, 33], Additional file 1: Fig. S1A, B). We sought to investigate the neuroinflammatory response in the neonatal brain post-H-I and ask whether metformin regulated microglia and astrocyte activation in this model. Experiments were performed in GFAP-GFP mice. On P8, mice underwent H-I, which consisted of the ligation of the left common artery, followed by a one-hour rest period and subsequent one-hour period of hypoxia. One hour following the entirety of the H-I injury, mice were assessed on the righting reflex to confirm that the H-I injury resulted in described impairments in motor function [10]. As expected, H-I-injured mice showed a significant increase in the latency to prone (*p* = 7.34 × 10^–5^) (Additional file 2: Fig. S2A, B). We characterized the neuroinflammatory cells, microglia and astrocytes, on P9, P12, P14 and P21, in control and H-I-injured mice, with and without metformin treatment from P9 to P14 (Fig. 1A). Our analyses focused on brain regions associated with motor function, namely, the motor cortex and striatum, based on previous work demonstrating that H-I-induced motor impairments are rescued by metformin administration (Dadwal et al., [33], Additional file 1: Fig. S1A, B). We also examined the subependymal zone (SEZ) which contains neural precursor cells (NPCs) that are known to be activated following H-I with enhanced proliferation and migration to the injured parenchyma where they give rise to new neurons and glia following metformin treatment [10].

We first assessed the total numbers of DAPI labelled cells per unit area in each of the cortex, striatum and SVZ of brains from the following groups of mice: Sham (uninjured), Sham + metformin, H-I only and H-I + metformin. We observed no difference in cell density across regions over time (P9, P12, P14 and P21) or between groups (Additional file 2: Fig. S2C-E). Next, we assessed the numbers of Iba1 + microglia in each region at P9, 12, 14 and 21. We found a significant increase in Iba1 + microglia in H-I-injured brains relative to Shams at P12 in both the cortex (2.4-fold increase; *p* = 0.0063) (Fig. 1B–F) and striatum (2.7-fold increase; *p* = 0.032) (Fig. 1 H–L) which returned to control levels by P14 (Fig. 1B–M). There was no change in numbers of Iba + microglia in the SEZ following H-I (Fig. 1N–R). The increased numbers of microglia after H-I in the cortex and striatum were coincident with a significant increase in proliferating microglia (Iba1 + EdU + cells) in the cortex (*p* = 0.0064) and striatum (*p* = 0.0085) relative to Shams (Fig. 1G, M). Notably, in all regions, metformin treatment alone did not alter the numbers or the proliferation of microglia when compared to Sham-treated mice (Fig. 1F, G, L, M, R). Moreover, metformin treatment after H-I did not alter the magnitude of the microglia expansion (2.1-fold increase in the cortex, *p* = 0.073; 3.0-fold increase in the striatum, *p* = 0.29) of microglia when compared to H-I only-injured mice (Fig. 1F, M). Similarly, the percentage of Iba1 + Edu + cells was unchanged between H-I and H-I + metformin-treated mice in the cortex and striatum (Fig. 1G, M). Hence, H-I results in a temporally and a regionally distinct expansion in microglia, irrespective of metformin treatment.

### Microglia morphology and protein expression is modified following H-I in the presence of metformin

**Fig. 2:**
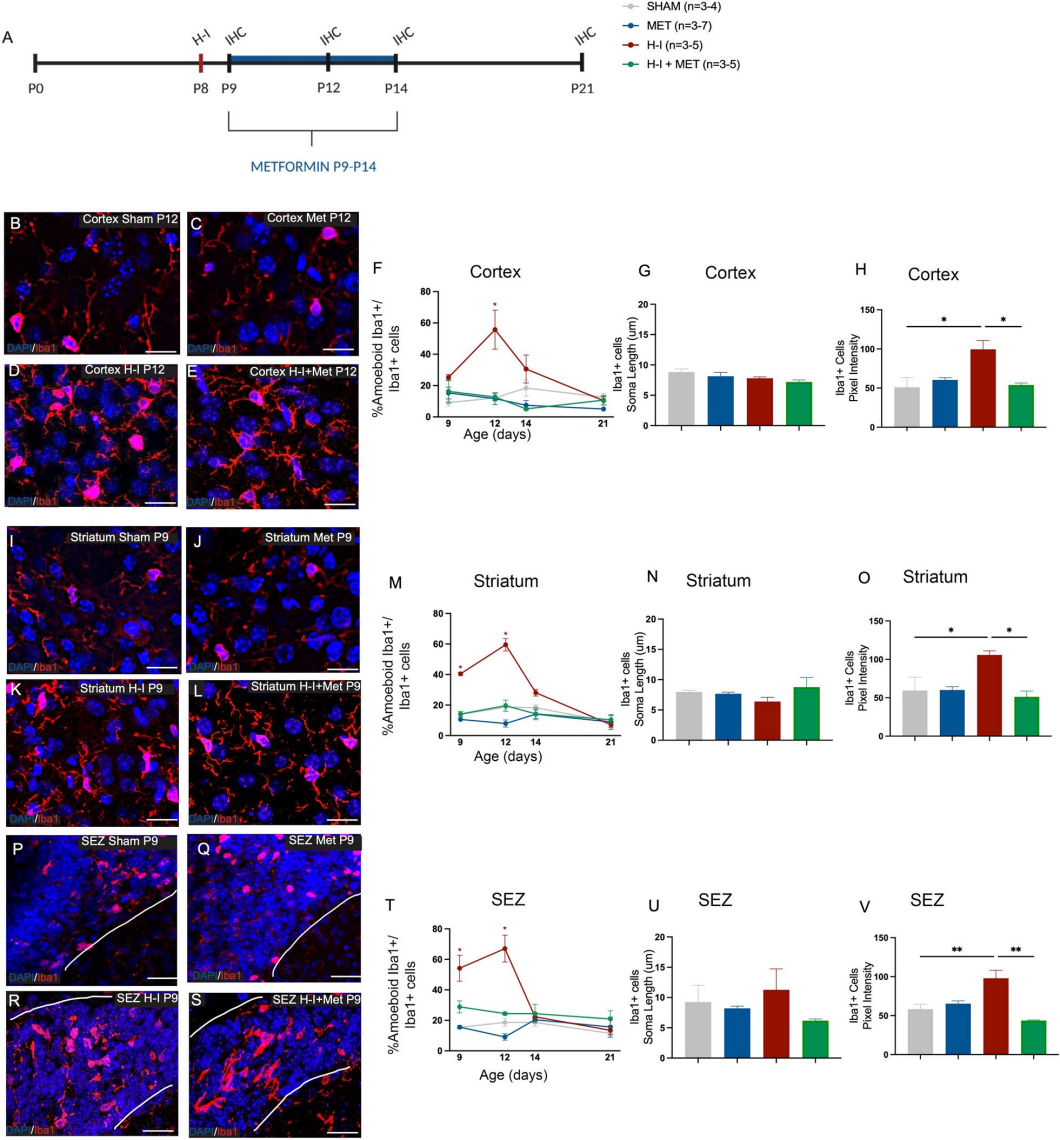
Metformin reduces amoeboid microglia after H-I. **A **Experimental timeline. Mice received H-I on P8 and metformin administration from P9 to P14. Immunohistochemistry (IHC) was performed on P9, P12, P14 and P21. **B **Representative image of microglia (Iba1 +) at P12 in the cortex in Sham mice (×40 magnification). The white box is a magnification depicting ramified microglia morphology. **C **Representative image of microglia (Iba1 +) at P12 in the cortex in Met-treated mice (×40 magnification). The white box is a magnification depicting ramified microglia morphology. **D **Representative image of microglia (Iba1+, red) at P12 in the cortex in H-I mice (×40 magnification). The white box is a magnification depicting amoeboid morphology. Image is representative of the first time point of change in morphology. **E **Representative image of microglia (Iba1 +) at P12 in the cortex in H-I + MET mice (×40 magnification). The white box is a magnification depicting ramified microglia morphology. **F **Quantification of the amoeboid microglia (Iba1 +) in the cortex across time points and groups. The percentage of amoeboid microglia was significantly increased after H-I at P12 relative to Sham (12.67 ± 2.10% amoeboid Iba1 + cells in Sham mice vs. 55.68 ± 12.43%in H-I-injured mice, *p* = 0.00011). This increase was prevented by metformin treatment (11.62 ± 3.79% amoeboid Iba1 + cells in Met-treated mice vs. 12.69 ± 2.41% amoeboid Iba1 + cells in H-I + Met-treated mice, *p* = > 0.99). **G** Quantification of the soma length of microglia (Iba1 +) in the cortex across groups. There was no difference in soma length across treatment groups (8.82 ± 0.50 µm soma length in Sham vs. 7.81 ± 0.22 µm after H-I; 8.14 ± 0.62 µm soma length in Met vs. 7.19 ± 0.34 µm in H-I + Met, *p* = 0.15). **H** Quantification of the pixel intensity of microglia (Iba1+) in the cortex across groups. There was a significant increase in the intensity of Iba1+ microglia after H-I relative to Sham (51.09 ± 12.38 pixels in Sham vs. 99.6 ± 11.27 pixels after H-I, p = 0.017). There was a significant decrease in Iba1 + microglia after H-I + Met relative to H-I (99.6 ± 11.27 after H-I vs. 54.0 ± 2.40 pixels in H-I + Met, *p* = 0.023) but no difference in intensity between Met and H-I + Met (60.4 ± 2.80 pixels in Met vs. 54.0 ± 2.40 pixels in H-I + Met, *p* = 0.95). Representative image of amoeboid microglia (Iba1+) at P9 in the striatum in Sham mice (×40 magnification). Image is representative of the first time point of change in morphology. **I** Representative image of amoeboid microglia (Iba1+) at P9 in the striatum in Met-treated mice (×40 magnification). Image is representative of the first time point of change in morphology. **J** Representative image of amoeboid microglia (Iba1 +) at P9 in the striatum in H-I mice (×40 magnification). Image is representative of the first time point of change in morphology. **K** Representative image of amoeboid microglia (Iba1 +) at P9 in the striatum in H-I + MET mice (×40 magnification). **L** Quantification of the amoeboid microglia (Iba1+) in the striatum across time points and groups. **M** The percentage of amoeboid microglia was significantly increased after H-I at P9 relative to Sham (13.84 ± 1.9% amoeboid Iba1 + cells in Sham mice vs. 40.46 ± 0.97% in H-I-injured mice, *p* = 9.00 × 10^–8^). This increase was prevented by HI + Met (10.59 ± 1.32% amoeboid Iba1 + cells in Met-treated mice vs. 14.11 ± 1.68% amoeboid Iba1 + cells in H-I + Met-treated mice, *p* = 0.999 relative to Met and *p* = relative to H-I + Met). The percentage of amoeboid microglia was significantly increased after H-I at P12 relative to Sham (18.61 ± 2.53%, amoeboid Iba1 + cells in Sham mice vs. 59.48 ± 4.05% in H-I-injured mice, *p* = 1.87 × 10^–10^). This increase was prevented by HI + Met (7.89 ± 2.36% amoeboid Iba1 + cells in Met-treated mice vs.19.57 ± 3.67% amoeboid Iba1 + cells in H-I + Met-treated mice, *p* = 0.99).**N** Quantification of the soma length of microglia (Iba1 +) in the striatum across groups. There was no difference in soma length across treatment groups (7.98 ± 0.25 µm cell soma length in Sham 6.39 ± 0.68 µm after H-I; 7.68 ± 0.26 µm soma length in Met vs. 8.75 ± 1.60 µm in H-I + Met, *p* = 0.36). **O **Quantification of the pixel intensity of microglia (Iba1 +) in the striatum across groups. There was a significant increase in the intensity of Iba1 + microglia after H-I relative to Sham (59.5 ± 17.37 pixels in Sham vs. 105.7 ± 5.29 pixels after H-I, *p* = 0.046). There was no difference in pixel intensity after H-I + Met relative to Met (60.1 ± 5.29 pixels in Met vs. 105.7 ± 5.29 pixels after H-I, *p* = 0.92), but Met treatment significantly decreased in the intensity of Iba1 + microglia after injury (105.7 ± 5.29 pixels in H-I vs. 51.2 ± 7.51 pixels in H-I + Met, *p* = 0.021). Representative image of amoeboid microglia (Iba1 +) at P9 in the SEZ in Sham mice (×40 magnification). Image is representative of the first time point of change in morphology. Representative image of amoeboid microglia (Iba1 +) at P9 in the SEZ in Met-treated mice (×40 magnification). Image is representative of the first time point of change in morphology. Representative image of amoeboid microglia (Iba1 +) at P9 in the SEZ in H-I mice (×40 magnification). Image is representative of the first time point of change in morphology. Representative image of amoeboid microglia (Iba1 +) at P9 in the SEZ in H-I + MET mice (×40 magnification). Quantification of the amoeboid microglia (Iba1 +) in the SEZ across time points and groups. The percentage of amoeboid microglia was significantly increased after H-I at P9 relative to Sham (15.43 ± 1.54% amoeboid Iba1 + cells in Sham mice vs. 54.16 ± 8.53% in H-I-injured mice, *p* = 8.85 × 10^–5^). This increase was prevented by HI + Met (15.54 ± 1.04% amoeboid Iba1 + cells in Met-treated mice vs. 22.87 ± 3.93% amoeboid Iba1 + cells in H-I + Met-treated mice, *p* = 0.841). The percentage of amoeboid microglia was significantly increased after H-I at P12 relative to Sham (18.62 ± 2.5%, amoeboid Iba1 + cells in Sham mice vs. 67.04 ± 8.81% in H-I-injured mice, *p* = 1.06 × 10^–5^) and again, this increase was prevented by HI + Met (9.13 ± 2.07% amoeboid Iba1 + cells in Met-treated mice vs. 24.44 ± 1.02% amoeboid Iba1 + cells in H-I + Met-treated mice, *p* = 0.763).Quantification of the soma length of microglia (Iba1 +) in the SEZ across groups. There was no difference in soma length across treatment groups (9.24 ± 2.79 µm soma length in Sham vs. 11.26 ± 3.46 µm after H-I; 8.21 ± 0.37 µm soma length in Met vs. 6.17 ± 0.30 µm soma length in H-I + Met, *p* = 0.48). **V **Quantification of the pixel intensity of microglia (Iba1 +) in the SEZ across groups. There was a significant increase in the intensity of Iba1 + microglia after H-I relative to Sham (58.3 ± 16.21 pixels in Sham vs. 98.0 ± 10.08 pixels after H-I, *p* = 0.0084). There was no difference in pixel intensity after H-I + Met relative to Met (65.3 ± 3.6 pixels in Met vs. 51.2 ± 7.51 pixels in H-I + Met, *p* = 0.14) but Met treatment significantly decreased in the intensity of Iba1 + microglia after injury (98.0 ± 10.08 pixels in H-I vs. 43.7 ± 0.79 pixels in H-I + Met, *p* = 0.0012). *n* = 3–5 mice per group. Data presented as mean ± SEM. Scale Bar: 10um. Unit area (cortex, striatum): 3x0.105 mm^2^. Unit area (SEZ): 3x0.045mm^2^. Statistics: (D, G, J) Two-way ANOVA. *p≤0.050

While the numbers of microglia were not modified by metformin treatment, we predicted that changes in the activation state of the microglia would be seen based on metformin’s anti-inflammatory effects after injury [11, 21, 32, 38, 57﻿]. We examined two key features of the microglia activation after injury: morphology (i.e. amoeboid phenotype) and CD68 protein expression, a marker of phagocytosis. We quantified the relative percentage of Iba1 + microglia with an amoeboid phenotype, as defined by a rounded cell body morphology with a lack of processes extending from the soma. In the cortex, H-I increased the proportion of amoeboid microglia at P12 (43% increase from Sham; *p* = 8.43 × 10^–5^), a time when increased numbers of microglia were observed (Fig. 2B–E, F). A similar pattern of microglia activation was observed in the striatum, with significantly increased proportions of amoeboid microglia post-H-I and this activation was seen at both P9 (27% increase from Sham; *p* = 8.94 × 10^–9^) and P12 (41% increase from Sham; *p* = 1.87 × 10^–11^) (Fig. 2I–L, M). Interestingly, while no changes in the numbers of Iba1 + cells were seen in the SEZ after H-I, we observed a significant increase in the relative percent of amoeboid microglia as early as P9 (38.73% increase from Sham; *p* = 8.85 × 10^–5^) and at P12 (48.42% increase from Sham; *p* = 1.06 × 10^–5^) (Fig. 2P–S, T). Strikingly, H-I + metformin-treated mice showed a significant reduction in amoeboid microglia in the cortex at P12 (43% decrease from H-I; *p* = 0.00011), back to control (Sham) values (Fig. 2E, F). The same was true in the striatum, at P9 (26% decrease from H-I; *p* = 8.49 × 10^–7^) and P12 (40% decrease from H-I; *p* = 3.66 × 10^–10^), where metformin treatment after H-I resulted in a reduction in amoeboid microglia back to control (Sham injured) levels (Fig. 2L, M). Identical to what was observed in the parenchyma, H-I + metformin-treated mice had significantly reduced SEZ amoeboid microglia at P9 (25% decrease from H-I; p = 0.0052) and P12 (43% decrease from H-I; p = 0.00012) that was not significantly different from Shams (Fig. 2S, T). We did not observe a difference in the soma size of Iba1 + cells between treatment groups in any region assessed (cortex, *p* = 0.15; striatum, *p* = 0.36; SEZ, *p* = 0.48) (Fig. 2G, N, U); however, we did observe a significant increase in the intensity of the Iba1 + microglia after H-I that was consistent across brain regions (*p* = 0.0017 in the cortex, *p* = 0.046 in the striatum, and *p* = 0.0084 in the SEZ) relative to sham animals (Fig. 2H, O, V). This finding is consistent with previous reports of cerebral ischaemia increasing the intensity of Iba1 + microglia at acute time-points after injury [25]. Interestingly, metformin treatment significantly reduced the intensity of Iba1 + microglia to control levels (cortex, *p* = 0.023,striatum, *p* = 0.021; SEZ, *p* = 0.0012) (Fig. 2H, O,V). Hence, metformin administration reduces microglia activation post-H-I.

We further assessed microglia activation using CD68 protein expression, a marker of microglia phagocytosis, using immunohistochemistry (Additional file 3: Fig. S3). Coincident with the times we observed an increase in amoeboid microglia following H-I, we found a significant increase in the proportions of Iba + CD68 + /Iba1 + cells in the cortex at P12 (35% increase from Sham; *p* = 0.013) (Additional file 3: Fig. S3A–F); striatum at P9 (38% increase from Sham; *p* = 0.042) and P12 (46% increase from Sham; *p* = 0.0010) (Additional file 3: Fig. S3G–L); and SEZ at both P9 (46% increase from Sham; *p* = 0.0060) and P12 (47% increase from Sham; *p* = 0.023) (Additional file 3: Fig. S3M–R). Most striking, we observed a decrease in the proportion of Iba1 + CD68 + /Iba1 + cells in H-I + metformin-treated mice in all regions examined relative to H-I only mice (cortex, 32% decrease at P12, *p* = 0.022; striatum, 47% decrease at P9 and 42% decrease at P12, *p* = 0.013 and *p* = 0.002, respectively; SEZ, 53% decrease at P9 and 52% decrease at P12, *p* = 0.0024 and *p* = 0.013, respectively) (Additional file 3: Fig. S3F, K, L, Q, R). Together, these data demonstrate that metformin administration reduces the activation of microglia following H-I by reducing the presence of amoeboid, CD68 + expressing microglia.

**Fig. 3:**
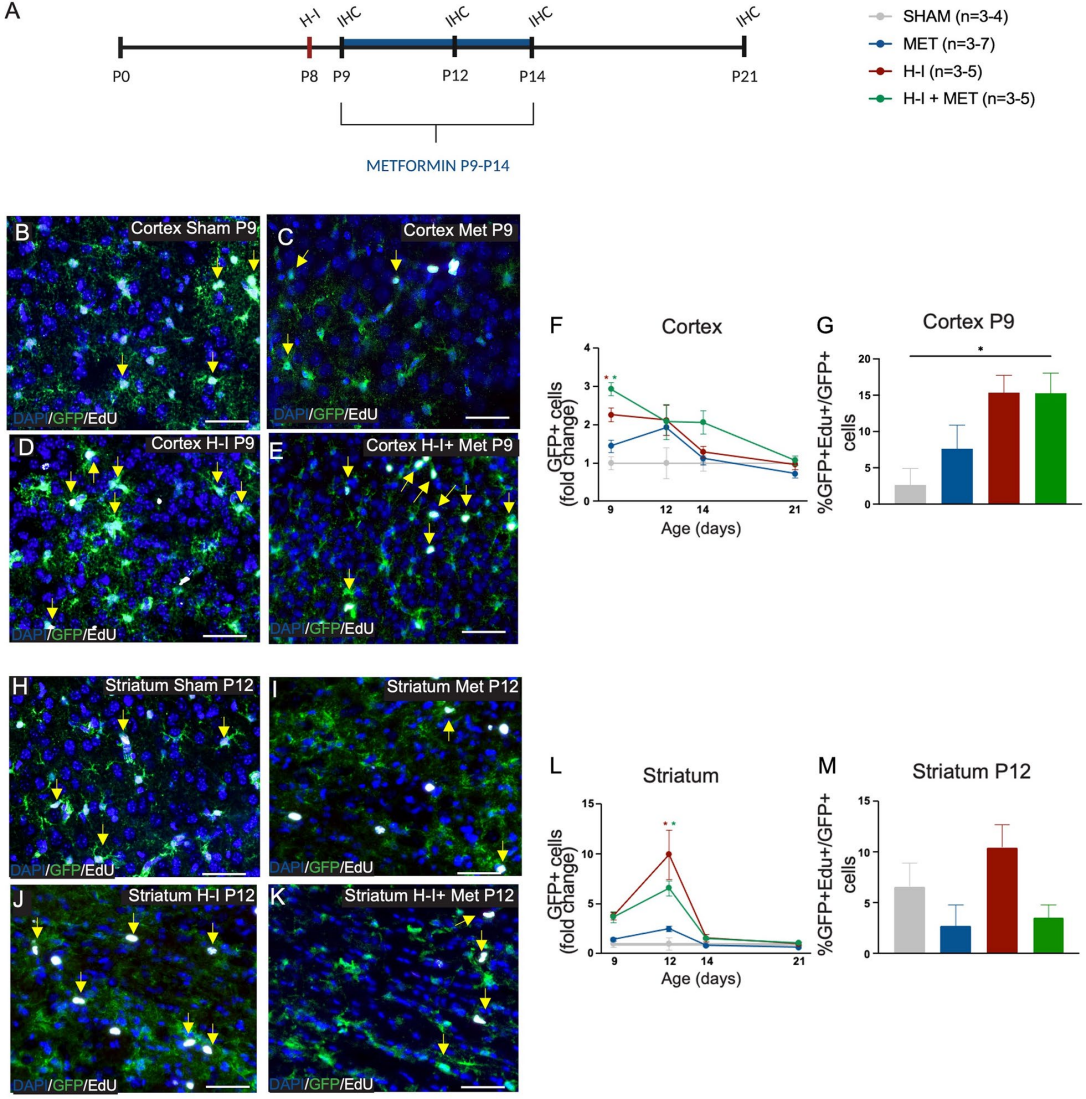
H-I, in the presence or absence of metformin, increases astrocyte numbers in the cortex and striatum. **A** Experimental timeline. Mice received H-I on P8 and metformin administration from P9-P14. Immunohistochemistry (IHC) was performed on P9, P12, P14 and P21. **B** Representative image of astrocytes (GFP+, green) and proliferating astrocytes (GFP + (green) EdU + (white) double-positive cells; yellow arrows) at P9 in the cortex in Sham (×20 magnification). **C** Representative image of astrocytes (GFP+, green) and proliferating astrocytes (GFP + (green) EdU + (white) double-positive cells; yellow arrows) at P9 in the cortex in Met-treated mice (×20 magnification). **D** Representative image of astrocytes (GFP +) and proliferating astrocytes (GFP + EdU+, yellow arrows) at P9 in the cortex after H-I (×20 magnification). **E** Representative image of astrocytes (GFP +) and proliferating astrocytes (GFP + EdU+, yellow arrows) at P9 in the cortex after H-I + Met (×20 magnification). **F** Quantification of the number of astrocytes (GFP +) in the cortex across time points and groups expressed as fold change. Astrocyte numbers were significantly increased after H-I at P9 relative to Sham (1.00 ± 0.17-fold change of GFP + cells in Sham mice vs. 2.27 ± 0.19 in H-I-injured mice, *p* = 0.0026). This increase was not prevented by HI + Met (1.45 ± 0.16-fold change of GFP + cells in Met-treated mice vs. 2.94 ± 0.17-fold change of GFP cells in H-I + Met-treated mice, *p* = 0.0044). Average number of GFP + cells/unit area: Sham = 27.94 ± 4.81; Met = 40.50 ± 4.57; H-I = 63.30 ± 5.17, H-I + Met = 82.00 ± 4.87. **G** Quantification of proliferating astrocytes (GFP + EdU + /GFP +) in the cortex at P9 across groups. Astrocyte numbers were significantly increased after H-I at P9 relative to Sham (2.64 ± 2.27% GFP + EdU + cells in Sham mice vs. 15.36 ± 2.37% in H-I-injured mice, *p* = 0.035). There was no significant difference in GFP + EdU + cells in Met-treated mice relative to and H-I + Met (7.63 ± 3.25% GFP + EdU + cells in Met-treated mice vs. 15.28 ± 2.77% GFP + EdU + cells in H-I + Met-treated mice, *p* = 0.27). **H** Representative image of astrocytes (GFP +) and proliferating astrocytes (GFP + EdU+, yellow arrows) at P12 in the striatum in Sham mice (×20 magnification). **I** Representative image of astrocytes (GFP+, green) and proliferating astrocytes (GFP + (green) EdU + (white) double-positive cells; yellow arrows) at P12 in the striatum in Met-treated mice (×20 magnification). **J** Representative image of astrocytes (GFP +) and proliferating astrocytes (GFP + EdU+, yellow arrows) at P12 in the striatum after H-I injury (×20 magnification). **K** Representative image of astrocytes (GFP +) and proliferating astrocytes (GFP + EdU+, yellow arrows) at P12 in the striatum after H-I + Met (×20 magnification). **L** Quantification of the number of astrocytes (GFP +) in the striatum across time points and groups expressed as fold change. Astrocyte numbers were significantly increased after H-I at P12 relative to Sham (1.00 ± 0.63-fold change of GFP + cells in Sham mice vs. 9.95 ± 2.46 in H-I-injured mice, *p* = 6.69 × 10^–9^). This increase was not prevented by HI + Met (2.49 ± 0.26-fold change of GFP + cells in Met-treated mice vs. 6.58 ± 0.74-fold change of GFP cells in H-I + Met-treated mice, *p* = 6.69 × 10^–9^). Average number of GFP + cells/unit area: Sham = 5.67 ± 3.56; Met = 14.09 ± 1.46; H-I = 56.38 ± 13.96, H-I + Met = 37.27 ± 4.18. **M** Quantification of proliferating astrocytes (GFP + EdU + /GFP +) in the striatum at P12 across groups. No significant differences were recorded across groups at P12 (*p* = 0.054). n = 3–7 mice per group. Data presented as mean ± SEM. Scale Bar: 50um. Unit area (cortex, striatum): 3 × 0.105 mm^2^. Statistics: (D, H) Two-way ANOVA; (E, I) One-way ANOVA. *p ≤ 0.050

### Astrocytes increase in numbers after H-I, with or without metformin, in the cortex and striatum

Astrocytes are responsive to brain injury and recent work in the adult brain has revealed that microglia play a role in astrocyte activation [30, 49]. Moreover, astrocytes in the adult brain are responsive to metformin in a model of Parkinson’s Disease [30, 49]. Based on these reports revealing the interplay between microglia and astrocytes and our observation that metformin regulates the microglia response post-H-I, we examined the numbers of GFAP expressing astrocytes in the cortex and striatum of GFAP-GFP mice in the same groups as described above (Fig. 3A. First, we examined the astrocyte response post-H-I only. In the cortex, we observed a significant increase in GFP + cells as early as P9 post-H-I (2.3-fold increase from Sham; *p* = 0.0026 and this was concomitant with an increase in GFP + EdU + /GFP + cells at P9 (*p* = 0.035) (Fig. 3B-G). In the striatum, GFP + cells were significantly increased at P12, the same time as microglia activation post-H-I (9.9-fold increase from Sham; *p* = 6.69 × 10^–9^); however, this increase in numbers was not coincident with increased EdU + GFP + /GFP + cells (Fig. 3H–M), suggesting that the proliferation occurred prior to the time of EdU injection at P12.

Metformin administration following H-I resulted in the same increase in GFP + cells at P9 in the cortex (1.5-fold increase from Met, *p* = 0.0044), with a trend towards an increase in proliferating astrocytes (8% increase from Met; *p* = 0.27) (Fig. 3B–G). In the striatum, H-I + metformin treatment resulted in increased numbers of GFP + cells (4.1-fold increase from Met; *p* = 6.69 × 10^–9^) (Fig. 3H–M). Again, this increase in numbers was not coincident with increased EdU + GFP + /GFP + cells, suggesting that the proliferation occurred prior to the time of EdU injection at P12. Taken together, these data suggest regional heterogeneity in terms of astrocyte activation and additionally, unlike what is observed in the adult injured brain, metformin administration does not dampen the astrocyte response in the neonatal brain.

### Plexxikon 5622 successfully ablates microglia in neonates without affecting motor behaviour

Towards the goal of determining the cellular basis for metformin’s positive effects on motor outcomes post-H-I and based on our findings that metformin significantly reduced microglia activation post-H-I, we asked if microglia ablation prior to H-I would result in improved functional outcomes. We sought to establish a microglia ablation paradigm in which microglia would be selectively depleted within the neonatal brain at the time of injury and coincident with the time when metformin administration leads to improved functional outcomes [10], Additional file 1: Fig. S1). We used a pharmacological approach to deplete microglia using Plexxikon 5622 (PLX) is a colony stimulating factor 1 (CSF1) receptor antagonist, which inhibits microglia survival (Riquer and Sollars 2020; [53]. We administered daily i.p. injections of PLX to neonatal mice starting on P8. The number of Iba1 + microglia was assessed in the cortex, striatum and SEZ at P15 (Additional file 4: Fig. S4A). Throughout this study, pup weight was monitored daily as a measure of overall health. PLX treatment had no effect on weight (Additional file 4: Fig. S4B). We found that one week of 50ug/g PLX treatment (P8–P15 resulted in a significant depletion of Iba1 + microglia in the cortex (5.5-fold decrease from vehicle; *p* = 0.033 (Additional file 4: Fig. S4C-F), striatum (3.3-fold decrease from vehicle; *p* = 0.0034) (Additional file 4: Fig. S4G-J) and SEZ (3.3-fold decrease from vehicle; *p* = 0.0038) (Additional file 4: Fig. S4K-N). In the SEZ, previous studies have shown that SEZ microglia can regulate the behaviour of SEZ neural stem cells by enhancing their proliferation [35, 46]. Hence, we predicted that the loss of microglia would reduce the number of neural stem cell-derived colonies (neurospheres) in vitro. The numbers of neurospheres were assessed at P15 following PLX treatment (when the greatest depletion of microglia is observed) and we observed no change in the numbers of neurospheres between vehicle- and PLX-treated mice (Additional file 4: Fig. S4O). Hence, microglia ablation in neonates does not result in changes in astrocytes and neural stem cell numbers.

**Fig. 4:**
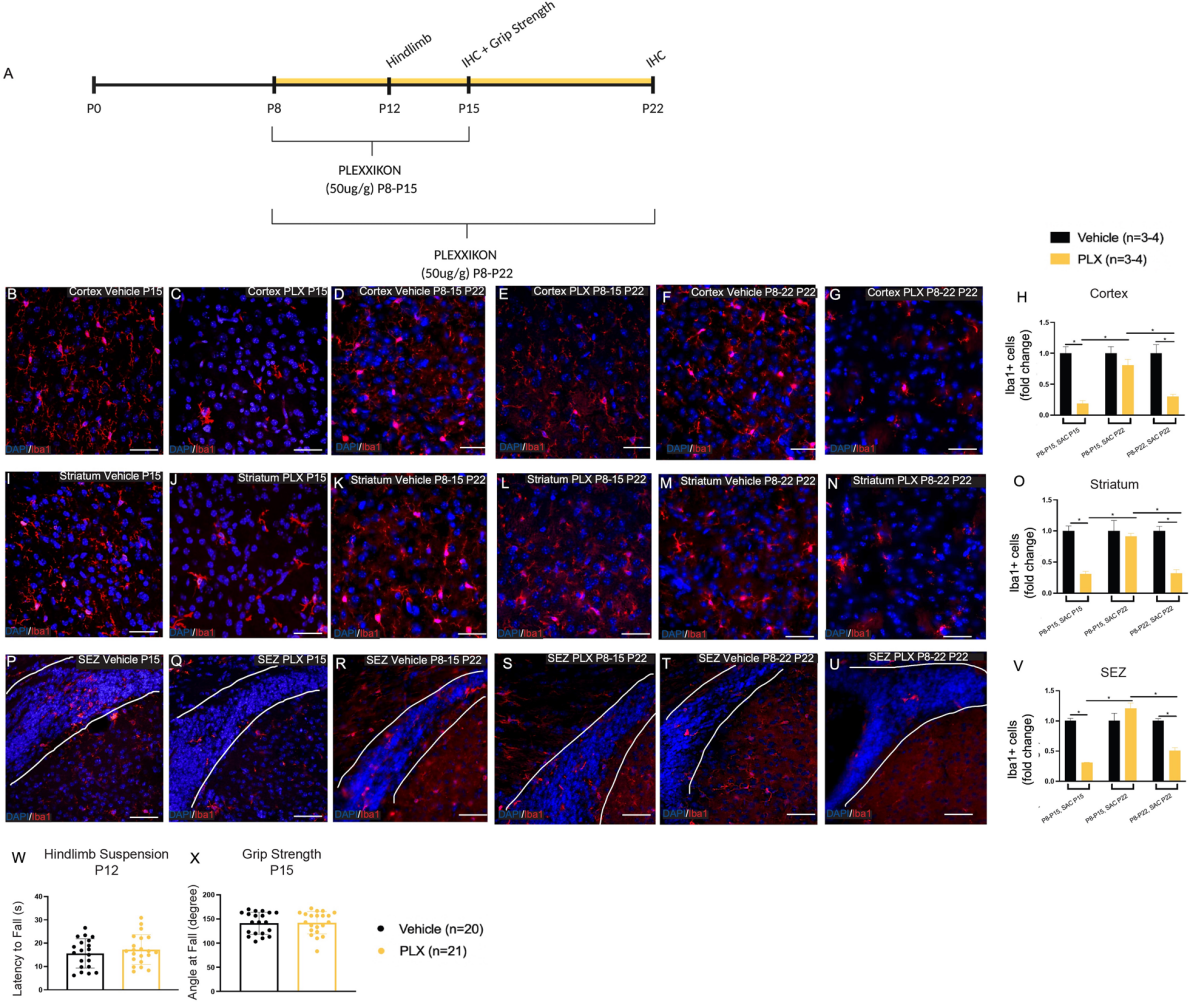
Treatment cessation following microglia ablation, which had no effects on astrocytes or sham motor behaviour, causes rapid microglia repopulation. **A** Experimental timeline. Mice received PLX administration from P8 to P15 (SAC on P15 or P22) or P8 to P22 (SAC on P22). Immunohistochemistry (IHC) was performed on P15 and P22. Hindlimb suspension was performed on P12 and Grip Strength on P15. **B** Representative image of microglia (Iba1+, red) at P15 in the cortex of vehicle-treated mice (×20 magnification). **C** Representative image of microglia (Iba1+, red) at P15 in the cortex of mice treated with PLX from P8 to P15 (×20 magnification). **D** Representative image of microglia (Iba1+, red) at P22 in the cortex of vehicle-treated mice (×20 magnification). **E** Representative image of microglia (Iba1+, red) at P22 in the cortex of mice treated with PLX from P8 to P15. **F** Representative image of microglia (Iba1+, red) at P22 in the cortex of vehicle-treated mice (×20 magnification). **G** Representative image of microglia (Iba1+, red) at P22 in the cortex of mice treated with PLX from P8 to P22. **H** Quantification of the number of microglia (Iba1 +) in the cortex across groups expressed as fold change. There was a significant decrease in microglia after PLX treatment relative to vehicle at P15 (1.00 ± 0.11-fold change of Iba1 + cells in vehicle-treated mice vs. 0.19 ± 0.05 in H-I-injured mice, *p* = 0.0008). There was no difference in microglia numbers between vehicle vs. PLX at P22 following treatment from P8 to P15 (1.00 ± 0.11-fold change of Iba1 + cells in vehicle-treated mice vs. 0.80 ± 0.10 in H-I-injured mice, *p* = 0.646). There was a significant decrease in microglia after treatment relative to vehicle at P22 following two weeks of treatment (PLX from P8 to P22) (1.00 ± 0.14-fold change of Iba1 + cells in vehicle-treated mice vs. 0.30 ± 0.04 in H-I-injured mice, *p* = 0.003). Average number of Iba1 + cells/unit area: P15 Vehicle = 67.17 ± 7.21; P15 PLX = 12.42 ± 3.2; P8-P15 Sac 22 Vehicle = 19.83 ± 3.06; P8-P15 Sac 22 PLX = 21.42 ± 2.69; P8-P22 Sac 22 Vehicle = 40.68 ± 5.60, P8-P22 Sac 22 PLX = 12.22 ± 1.49. **I** Representative image of microglia (Iba1 +) at P15 in the striatum of vehicle-treated mice (×20 magnification). **J** Representative image of microglia (Iba1 +) at P15 in the striatum of PLX-treated mice (×20 magnification). **K** Representative image of microglia (Iba1+, red) at P22 in the striatum of vehicle-treated mice (×20 magnification). **L** Representative image of microglia (Iba1+, red) at P22 in the striatum of mice treated with PLX from P8 to P15. **M** Representative image of microglia (Iba1+, red) at P22 in the striatum of vehicle-treated mice (×20 magnification). **N** Representative image of microglia (Iba1+, red) at P22 in the striatum of mice treated with PLX from P8 to P22. **O** Quantification of the number of microglia (Iba1 +) in the striatum across groups expressed as fold change. There was a significant decrease in microglia after PLX treatment relative to vehicle at P15 (1.00 ± 0.08-fold change of Iba1 + cells in vehicle-treated mice vs. 0.31 ± 0.04 in H-I-injured mice, *p* = 0.005). There was no difference in microglia numbers between vehicle vs. PLX at P22 following treatment from P8 to P15 (1.00 ± 0.17-fold change of Iba1 + cells in vehicle-treated mice vs. 0.91 ± 0.05 in H-I-injured mice, *p* = 0.982). There was a significant decrease in microglia after treatment relative to vehicle at P22 following two weeks of treatment (PLX from P8 to P22) (1.00 ± 0.08-fold change of Iba1 + cells in vehicle-treated mice vs. 0.32 ± 0.06 in H-I-injured mice, *p* = 0.005). Average number of Iba1 + cells/unit area: P15 Vehicle = 32.19 ± 2.65; P15 PLX = 9.83 ± 1.36; P8-P15 Sac 22 Vehicle = 17.83 ± 3.03; P8-P15 Sac 22 PLX = 16.25 ± 0.87; P8-P22 Sac 22 Vehicle = 20.33 ± 1.53, P8-P22 Sac 22 PLX = 6.44 ± 1.25. **P** Representative image of microglia (Iba1 +) at P15 in the SEZ of vehicle-treated mice (×20 magnification). **Q** Representative image of microglia (Iba1 +) at P15 in the SEZ of PLX-treated mice (×20 magnification). **R** Representative image of microglia (Iba1+, red) at P22 in the SEZ of vehicle-treated mice (×20 magnification). **S** Representative image of microglia (Iba1+, red) at P22 in the SEZ of mice treated with PLX from P8 to P15. **T** Representative image of microglia (Iba1+, red) at P22 in the SEZ of vehicle-treated mice (×20 magnification). **U** Representative image of microglia (Iba1+, red) at P22 in the SEZ of mice treated with PLX from P8 to P22. **V** Quantification of the number of microglia (Iba1 +) in the SEZ across groups expressed as fold change. There was a significant decrease in microglia after PLX treatment relative to vehicle at P15 (1.00 ± 0.04-fold change of Iba1 + cells in vehicle-treated mice vs. 0.31 ± 0.01 in H-I-injured mice, *p* = 0.001). There was no difference in microglia numbers between vehicle vs. PLX at P22 following treatment from P8 to P15 (1.00 ± 0.13-fold change of Iba1 + cells in vehicle-treated mice vs. 1.20 ± 0.09 in H-I-injured mice, *p* = 0.463). There was a significant decrease in microglia after treatment relative to vehicle at P22 following two weeks of treatment (PLX from P8 to P22) (1.00 ± 0.04-fold change of Iba1 + cells in vehicle-treated mice vs. 0.50 ± 0.05 in H-I-injured mice, *p* = 0.016). Average number of Iba1 + cells/unit area: P15 Vehicle = 20.03 ± 0.87; P15 PLX = 6.19 ± 0.10; P8-P15 Sac 22 Vehicle = 17.83 ± 3.03; P8-P15 Sac 22 PLX = 16.25 ± 0.87; P8-P22 Sac 22 Vehicle = 13.22 ± 0.48, P8-P22 Sac 22 PLX = 6.67 ± 0.67. **W** No significant differences between the latency to fall (s) on the hindlimb suspension task at P12 were recorded between vehicle- vs. PLX-treated mice (*p* = 0.42). **X** No significant differences between the angle at fall (degree) on the grip strength task at P15 were recorded between vehicle- vs. PLX-treated mice (*p* = 0.91). *n* = 3–4 mice per group for immunohistochemistry. *n* = 20–21 mice per group for behavioural testing. Data presented as mean ± SEM. Scale Bar: 50um. Unit area (cortex, striatum): 3 × 0.105 mm^2^. Unit area (SEZ): 3 × 0.045mm^2^. Statistics: (D, G, J) Two-way ANOVA; (K, L) Unpaired t-test. *p ≤ 0.050

Previous work in adult rodents has demonstrated that PLX treatment cessation results in a rapid recovery of microglia [14, 53]. To determine whether a similar observation occurred in the neonatal brain, the numbers of Iba1 + cells were assessed in mice that received 50 ug/g PLX treatment for one week (P8–P15) and brains were examined at P22 (a time when functional recovery was observed following H-I + metformin treatment) [10] (Fig. 4A–V). As shown in Fig. 4, complete repopulation of Iba1 + cells was observed by P22 after one week of treatment cessation in the cortex (Fig. 4D, E, H), striatum (Fig. 4K, L, O) and SEZ (Fig. 4R, S, V). Finally, in a separate cohort of mice, PLX was administered for two weeks (P8–P22) in order to maintain the Ib1 + depletion and/or achieve a greater depletion than recorded for one week of treatment, and the numbers of Iba1 + cells were quantified on P22. In all regions, Iba1 + cells were significantly depleted but not significantly different compared to one week of PLX treatment in the cortex (3.3-fold decrease from vehicle; *p* = 0.0033 (Fig. 4F–H), striatum (3.2-fold decrease from vehicle; *p* = 0.005) (Fig. 4M-O) and SEZ (2.0-fold decrease from vehicle; *p* = 0.016) (Fig. 4T–V). Hence, PLX treatment to neonatal pups leads to a significant loss of microglia, which are rapidly repopulated upon cessation of the PLX treatment.

Given the documented interplay between astrocytes and microglia, we asked whether microglia ablation resulted in an astrocyte response in the neonatal brain. We counted the numbers of astrocytes (GFP + cells) in the cortex and striatum following 1 or 2 weeks of PLX treatment and found no change in GFP + cells (Additional file 5: Fig. S5A). We next examined the activation profile of astrocytes after H-I and in the absence of microglia (i.e. H-I + PLX treatment). At P15, a time when H-I + PLX-treated mice had the greatest depletion of microglia and no behavioural deficits, there was no significant chance in astrocytes (GFP + cells) in the cortex (*p* = 0.12) (Additional file 5: Fig. S5B-F) or striatum (*p* = 0.22) (Additional file 5: Fig. S5G-K) relative to any treatment group. Hence, microglia ablation in neonates does not result in changes in astrocytes numbers.

While microglia ablation does not lead to functional impairments in uninjured adults, this has not been explored in neonates [14, 53]. We therefore asked if microglia ablation impacted motor behaviour using functional assays to evaluate gross motor function during and at the end of PLX treatment. PLX administration started on P8 and the hindlimb suspension test was performed at P12 and the grip strength test at P15 in vehicle- and PLX-treated mice. In both tasks, PLX-treated mice behaved similarly to vehicle-treated controls (Fig. 4W, X). These findings reveal that microglia ablation following PLX treatment does not result in motor impairments in uninjured mice.

### Microglia ablation mimics the effects of metformin treatment following H-I

**Fig. 5:**
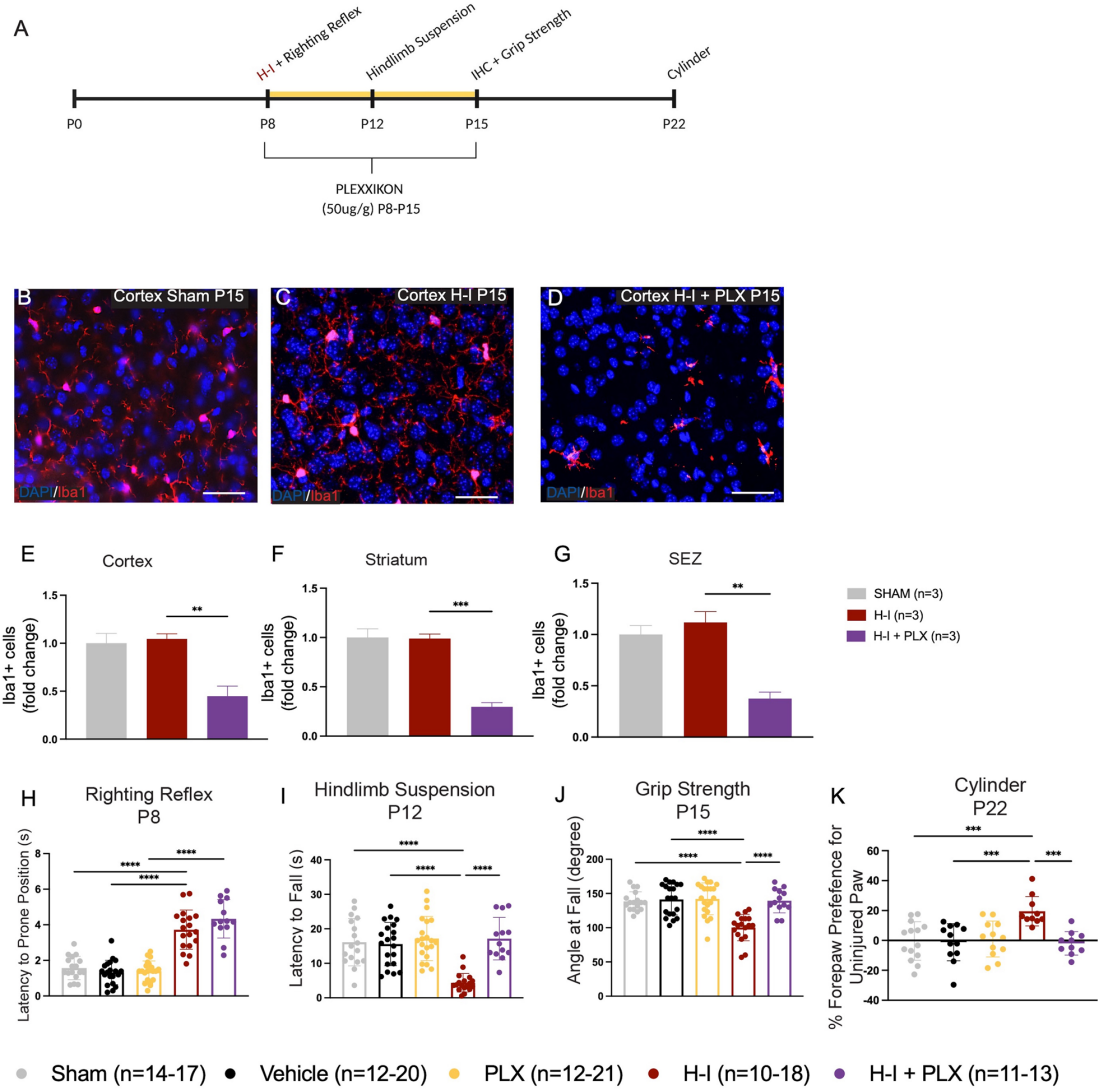
Microglia ablation affords protection against functional deficits after H-I. **A** Experimental timeline. H-I was performed on P8 and PLX administration from P8 to P15. IHC was performed on P15. Behaviour tasks were performed on P8 (righting reflex), P12 (hindlimb suspension), P15 (grip strength) and P22 (cylinder task). **B** Representative image of microglia (Iba1+, red) at P15 in the cortex of Sham mice (×20 magnification). **C** Representative image of microglia (Iba1+, red) at P15 in the cortex of H-I mice (×20 magnification). **D** Representative image of microglia (Iba1 +) at P15 in the cortex of H-I + PLX mice (×20 magnification). **E** Quantification of the number of microglia (Iba1 +) in the cortex across groups expressed as fold change. There was no difference in Iba1 + microglia after H-I at P15 (1.00 ± 0.10-fold change of Iba1 + cells/unit area in Sham vs. 1.05 ± 0.05-fold change of Iba1 + cells/unit area after HI) (p = 0.93). There was a significant decrease in Iba1 + microglia after H-I + PLX relative to H-I (1.05 ± 0.05-fold change of Iba1 + cells/unit area after H-I vs. 0.45 ± 0.11-fold change of Iba1 + cells/unit area after H-I + PLX) (*p* = 0.0076). Average number of Iba1 + cells/unit area: Sham = 67.17 ± 7.21; H-I = 48.11 ± 2.38; H-I + PLX = 20.56 ± 4.83. **F** Quantification of the number of microglia (Iba1 +) in the striatum across groups, expressed as fold change. There was no difference in Iba1 + microglia after H-I at P15 (1.00 ± 0.09-fold change of Iba1 + cells/unit area in Sham vs. 0.99 ± 0.04-fold change of Iba1 + cells/unit area after H-I) (*p* = 0.99). There was a significant decrease in Iba1 + microglia after H-I + PLX relative to H-I (0.99 ± 0.04-fold change of Iba1 + cells/unit area after H-I vs. 0.30 ± 0.04-fold change of Iba1 + cells/unit area after H-I + PLX) (*p* = 0.00060). Average number of Iba1 + cells/unit area: Sham = 32.19 ± 2.65; H-I = 31.11 ± 1.46; H-I + PLX = 9.33 ± 1.35. **G** Quantification of the number of microglia (Iba1 +) in the SEZ across groups, expressed as fold change. There was no difference in Iba1 + microglia after H-I at P15 (1.00 ± 0.09-fold change of Iba1 + cells/unit area in Sham vs. 1.12 ± 0.11-fold change of Iba1 + cells/unit area after H-I) (*p* = 0.62). There was a significant decrease in Iba1 + microglia after H-I + PLX relative to H-I (1.12 ± 0.11-fold change of Iba1 + cells/unit area after H-I vs. 0.25 ± 0.06-fold change of Iba1 + cells/unit area after H-I + PLX) (*p* = 0.0022). Average number of Iba1 + cells/unit area: Sham = 20.03 ± 0.87; H-I = 17.89 ± 1.68; H-I + PLX = 6.00 ± 1.00. **H** Latency to prone (s) on the righting reflex across groups. Significant increases were found after injury between Sham vs. H-I (*p* = 3.86 × 10^–10^), Vehicle vs. H-I (*p* = 1.59 × 10^–10^), and PLX vs. H-I + PLX (*p* = 1.58 × 10^–10^). There was no difference in the righting reflex between H-I vs. H-I + PLX (*p* = 0.26). **I** Latency to fall (s) on the hindlimb suspension across groups. Significant decreases were found after injury between Sham vs. H-I (*p* = 6.69 × 10^–7^), Vehicle vs. H-I (*p* = 7.95 × 10^–7^) and H-I vs. H-I + PLX (*p* = 5.19 × 10^–7^).** J** Angle at fall (degree) on grip strength across groups. Significant decreases were found after injury between Sham vs. H-I (*p* = 2.46 × 10^–6^), Vehicle vs. H-I (*p* = 1.37 × 10^–7^) and H-I vs. H-I + PLX (*p* = 7.03 × 10^–6^). **K** Forepaw preference for the uninjured paw on the cylinder test. H-I-injured mice are significantly impaired compared to Shams (-0.29 ± 3.44% preference for the un-impaired paw in Sham mice vs. 19.56 ± 3.12% in H-I-injured mice, *p* = 0.0009). PLX-treated (*p* = 0.99) and vehicle-treated mice (*p* = 0.99) did not perform differently than Shams. H-I + PLX are significantly improved compared to H-I only (-1.96 ± 2.48% preference for the un-impaired paw in H-I + Met mice vs. 19.56 ± 3.12% in H-I-injured mice, *p* = 0.0009) and were not significantly different from Shams (*p* = 0.99). *n* = 3 mice per group for immunohistochemistry. *n* = 6–21 mice per group for behavioural testing. Data presented as mean ± SEM. Scale Bar: 50um. Unit area (cortex, striatum): 3 × 0.105 mm^2^. Unit area (SEZ): 3 × 0.045mm^2^. Statistics: (D-J) One-way ANOVA. *p ≤ 0.050

To ask if the loss of microglia activation post-H-I was sufficient to reduce motor impairments following H-I, mice received H-I on P8 and PLX treatment until P15 (P8-15) (Fig. 5A). We performed immunohistochemistry for Iba1 + cells at P15 and found that similar to uninjured mice treated with PLX, there was a significant depletion in microglia in the cortex (2.4-fold decrease from H-I; *p* = 0.0076) (Fig. 5B–E), striatum (3.4-fold decrease from H-I; *p* = 0.00060) (Fig. 5F) and SEZ (3.0-fold decrease from H-I; *p* = 0.0022) (Fig. 5G) of mice given H-I and PLX. Hence, PLX treatment is sufficient to ablate microglia in both the uninjured and H-I injured neonatal brain.

Based on our findings that (1) metformin treatment after H-I reduces microglia activation and (2) metformin administration for 1 week post-H-I are sufficient to reduce motor impairments in the cylinder test on P22, we predicted that metformin’s anti-inflammatory effects on microglia may be mediating the improved functional outcomes seen after 1 week of metformin treatment (P8-15) and motor assessment on P22 [10] (Additional file 1: Fig. S1, Fig. 5A). Thus, we examined the motor outcomes following H-I + microglia ablation (PLX). Mice received H-I and PLX treatment from P8 to P15 to ablate microglia over the same time course that metformin administration is effective at reducing functional impairments in previous studies [10], Additional file 1: Fig. S1). Mice that received H-I only or H-I + PLX showed a significant increase in the latency to prone position in the righting reflex task when compared to uninjured mice (Fig. 5H). This validated the injury in the H-I-treated mice. On P12, the hindlimb suspension test was performed and only mice that had received H-I displayed a significant motor impairment (Fig. 5I). Similarly, the grip strength test on P15 revealed a deficit in H-I injured mice with no deficit observed in H-I + PLX mice which were not significantly different from uninjured groups (Fig. 5J). Most striking, H-I + PLX-treated mice were not impaired in the cylinder task at P22, identical to what is observed in H-I + metformin-treated mice that have metformin-mediated reduced microglia activation (Fig. 5K). Together, these findings reveal that a reduction in microglia activation, either through metformin treatment or ablation, is sufficient to eliminate the motor impairments that result following neonatal H-I injury.

## Discussion

In this study, we characterized the spatiotemporal response of microglia and astrocytes, in the neonatal brain following H-I, and modified microglia activation using two paradigms (Metformin and PLX). First, we asked whether the drug metformin, which is known to have pleiotropic effects on cells in the CNS, impacts microglia activation, with the goal of uncovering the cellular targets that underlie the beneficial outcomes observed following neonatal stroke and metformin treatment. Our findings reveal that microglia and astrocyte activation are regionally and temporally distinct within the injured brain and further that microglia activation is reduced during the time that metformin is administered post-H-I. We next developed a novel microglia ablation paradigm (PLX treatment) in neonatal mice and confirmed that the loss of microglia in the early post-natal brain is sufficient to protect against functional impairments following H-I. We found that reducing the activation of microglia—demonstrated by reduced proliferation, amoeboid morphology, CD68 expression and total numbers of microglia—through metformin treatment or microglia ablation resulted in a complete lack of motor impairments following H-I. The microglia ablation timeline mimicked the metformin administration that resulted in improved functional outcomes, implicating microglia as an important target for promoting recovery following neonatal injury.

Our study investigated the effects of modifying the activation state of microglia, as measured by proliferation, morphology and protein expression, and the impact on behavioural outcomes. A number of reports have shown that metformin has anti-inflammatory properties and can improve the microenvironment after injury by modulating the release of cytokines [15, 65]. Delivery of metformin has been shown to inhibit the inflammatory response 24 h post-injury in the early post-natal rodent brain and there have been reports of fewer Iba1 + microglia and GFAP + astrocytes in the cortex of after H-I + Metformin treatment compared to after HI-alone [15]. Interestingly, decreased expression of IL-6 and TNF-α was observed in the cortex of injured brains after metformin treatment and it was shown to be attenuated by the suppression of the TLR4/NF-κB signaling pathway, and notably, TLR4 is widely expressed on microglia [61]. Additional support for reduced levels of TNF-α, and IL-6, in addition to IL-1β, comes from studies of chronic metformin pre-treatment in a rodent model of adult stroke [65]. Metformin treatment ameliorated the microglial and astrogliotic response observed in mice that received stroke only [65]. Further, microglia numbers, pro-inflammatory morphology and expression of CD68 were also reduced after neonatal H-I when metformin treatment was delayed post-stroke [33]. Taken together, this suggests that understanding the factor(s) that modify the microenvironment and regulate microglia behaviour is potential targets and important considerations for brain repair strategies.

Moreover, the effects of metformin pre-treatment on microglia in the neurogenic niche, when metformin has been given prior to juvenile cranial radiation, has also revealed reduced microglia activation as measured by microglia numbers and CD68 expression [11]. Reduced microglia activation was measured using morphology and protein expression (CD68) and we observed a rapid increase in amoeboid microglia after H-I, which is consistent with previous studies [6, 18]. Both measures of activation were significantly reduced following metformin treatment. Interestingly, astrocytes, another key player in the neuroinflammatory response, were similarly activated by H-I and did not show a reduction in numbers in the presence of metformin. More recent work has shown that pro-inflammatory cytokines released by microglia are required for astrocytes activation [30], hence, we had predicted that astrocyte activation would occur after the microglia activation following H-I. This prediction was not supported by our findings, but instead, we observed a regionally distinct astrocyte activation that was earlier than microglia activation in the cortex of neonatal injured mice. These findings may highlight the differences between the neonatal and mature injured brain in response to injury.

We tested the hypothesis that neonatal H-I would trigger a microglia response and that metformin would dampen this response. Our observation that metformin did not reduce the microglia population after H-I was not predicted based on reports that metformin administration in the sub-acute period following early post-natal injury [15] reduces the absolute numbers of microglia. The different injury models and species (rat versus mouse) may account for the difference. In addition, the age of the mice at the time of injury, treatment and when the microglia are assessed can impact the outcomes observed. With regard to age, for example, microglia isolated from neonatal (2 days old) and weaned mice (21 days old) after LPS treatment revealed that only neonatal microglia showed increased mRNA expression of inflammatory markers, suggesting that microglia are much more responsive to injury in the neonate than in older animals [9]. These age-related differences are further supported by the fact that following H-I and sub-acute metformin treatment, the absolute numbers of microglia were decreased at P28 (i.e. adult microglia) [33]. The observed change in microglia and astrocyte numbers was not reflected in a change in the total number of DAPI + cells. This is consistent with concomitant with increases in other cell populations after metformin treatment, such as increased numbers of neurons and oligodendrocytes, as previously reported following neonatal stroke metformin administration [10]. Hence, our finding that H-I and metformin alter proliferation at early times post-H-I in mice and may reflect age- and species-dependent aspects of the neuroinflammatory process.

Metformin significantly reduced the numbers of amoeboid microglia after H-I. Microglia from H-I and metformin-treated brains failed to display an amoeboid phenotype which could be explained by (1) metformin preventing microglia from taking on an amoeboid phenotype or (2) H-I inducing the amoeboid phenotype and metformin reverts the activated microglia to their "resting" ramified phenotype. Given the early time point after injury at which we recorded a change in morphology after H-I (i.e. 1 day post-injury), our data support the hypothesis that metformin prevents the amoeboid phenotype. Microglia shape has been shown to be dependent on the Par1b/MARK2 pathway [13] (i.e. polarity-regulating kinase partitioning-defective pathway 1b/microtubule affinity-regulating kinase 2). Interestingly, Par1 activates NF-kb in lung endothelial cells following thrombin stimulation in vitro [7, 44]. Accordingly, metformin may act on the Parb/MARK 2 and NF-kb pathway to inhibit microglia from becoming amoeboid. Another role of metformin is to alter the inflammatory profiles of microglia (i.e. between pro-inflammatory and anti-inflammatory states) [32].﻿ Along these lines, metformin treatment after MCAO resulted in decreased staining for CD16/32 (pro-inflammatory) and increased staining for CD206 (anti-inflammatory at 14 days when compared to saline controls [26]. Interestingly, the cellular response to metformin is age dependent as have been seen with neural precursor cells [48] as well as with microglia [28] where metformin treatment led to increased proportions of CD206 + microglia and importantly, altered microglia morphology by increasing the number and complexity of their ramifications [28].

While others have examined the effects of different CSF1R inhibitors (BLZ945, PLX3397) on microglia in postnatal mice [36, 62], as well as in the context of H-I injury [62], our study builds on a study of PLX5622 in postnatal rats [47]. We have investigated the effects of PLX5622-mediated microglia depletion in neonatal mice on motor behaviours, in uninjured and stroke injured neonatal mice. Consistent with previous work, we observed depletion of microglia following the delivery of the CSF1R inhibitor and eventual repopulation after delivery of PLX5622 ceased [36]. Importantly, our experiments have shown that one week of Plexxikon 5622 treatment is sufficient to cause significant (~ 80%) depletion of microglia in the cortex, striatum and SEZ, without affecting astrocytes, NPCs or motor behaviour. Although microglia ablation has shown no motor or cognitive deficits in adults, we hypothesized that this may not be the same for neonates given the importance of microglia in early postnatal development [22, 43, 54, 60]. The effect of microglia ablation on fine motor and cognitive development in neonates remains unknown and more research is needed. Further highlighting the differences between adult and neonatal microglia ablation paradigms, Bellver-Landette and colleagues (2019) demonstrated worsened tissue and functional outcomes in the absence of microglia in the context of adult spinal cord injury [5]. Herein, we have shown that microglia ablation after H-I results in a lack of functional impairment on a battery of motor tests, which was correlated with a lack of functional impairment following metformin treatment after H-I. Therefore, we propose that metformin prevents microglia activation, and this is sufficient to prevent motor deficits following neonatal H-I.

## Conclusion

Our data reveal that dampening or ablating the early microglia response (i.e. within the first week post-H-I) is sufficient to protect against H-I-induced functional deficits. The findings are consistent with the conclusion that metformin-mediated reduction in microglia activation following H-I results in improved functional outcomes. This study further characterizes the pleiotropic effects of metformin and identifies microglia as a target to improve outcomes after neonatal stroke. Future investigation of strategies to assess microglia activation, such as assessing changes in inflammatory factors and using complementary ablation models (for example, CX3CR1-CreER x iDTR reporter mouse line),﻿ will contribute to our growing understanding of the cell mechanisms that underlies microglia-mediated recovery. Modulation of microglia activation states and exploring microglia heterogeneity remain important future considerations for novel strategies for brain repair.

## Supplementary Information

**Additional file 1: Figure S1.** H-I-injured mice that receive metformin treatment do not display a forepaw preference on the cylinder task. A Experimental timeline. Mice receive H-I injury on P8. Metformin was administered from P9 to P15. The cylinder test is performed on P22. B Forepaw preference for the uninjured paw on the cylinder test. Sham mice do not show a paw preference. H-I injured mice are significantly impaired compared to Shams (-0.29±3.44% preference for the un-impaired paw in Sham mice vs. 20.77±3.21% in H-I-injured mice) (p=0.0004). H-I+MET are significantly improved compared to H-I only (1.42±4.50% preference for the un-impaired paw in H-I+Met mice, p=0.012) and not significantly different from Shams (p=0.95). n=6–14 mice per group. Data presented as Mean ± SEM. Statistics: (B) One-way ANOVA. *p<0.050.

**Additional file 2: Figure S2.** H-I injury leads to behavioural impairments but no change in cell density in the cortex, striatum and SEZ. A Experimental timeline. Mice received H-I on P8 and Metformin administration from P9 to P14. Immunohistochemistry (IHC) was performed on P9, P12, P14 and P21. Righting reflex was performed on P8 at one hour post H-I. B H-I results in increased latency to perform the righting reflex test relative to Sham (1.26±0.16s latency in Sham mice vs. 7.63 ± 1.10s in H-I-injured mice, p=7.34x10-5) on P8 when performed 1 hour post-injury. C H-I, Metformin and H-I+MET treatments do not affect the total number of DAPI+ cells in the cortex. D H-I, Metformin and H-I+MET treatments do not affect the total number of DAPI+ cells in the striatum. E H-I, Metformin and H-I+MET treatments do not affect the total number of DAPI+ cells in the SEZ. n=3 mice per group for immunohistochemistry. n=11–16 mice per group for behavioural testing. Data presented as mean ± SEM. Unit area (cortex, striatum): 3x0.105 mm2. Unit area (SEZ): 3x0.045mm2. Statistics: (B) Unpaired t-test; (C-E) Two-way ANOVA. *p<0.050.

**Additional file 3: Figure S3.** Metformin treatment reduces CD68 expression in microglia after H-I. A Experimental timeline. H-I was given onP8 and metformin administration from P9 to P14.Immunohistochemistry (IHC) was performed on P9, P12, P14 and P21. B Representativeimage of activated microglia (Iba1+ (Red) CD68+ (Cyan) double-positive cells) atP12 in the cortex in Sham mice (20x magnification). Image is representative of thefirst time point of activation. C Representative image of activated microglia (Iba1+(Red) CD68+ (Cyan) double-positive cells) at P12 in the cortex after in Met-treatedmice H-I (20x magnification). Image is representative of the first time point ofactivation. D Representative image of activated microglia (Iba1+ (Red) CD68+ (Cyan)double-positive cells) at P12 in the cortex after H-I (20x magnification). Imageis representative of the first time point of activation. E Representative imageof activated microglia (Iba1+CD68+) at P12 (first time point of activation) in thecortex after H-I+MET (20x magnification). Image is representative of the first timepoint of activation. F Quantification of Iba1+CD68+ in the cortex at P12 acrossgroups, expressed as percentage per unit area. There was a significant increaseof Iba1+CD68+ microglia after H-I relative to Sham (26.74 ± 1.24% Iba1+CD68+ cellsin Sham mice vs. 61.70 ± 6.89%in H-I-injured mice, p=0.013). This increase in Iba1+CD68+cells was prevented by HI+Met (25.22± 2.23 % Iba1+CD68+ cells in Met-treated micevs. 29.84 ± 9.30% Iba1+CD68+ cells in H-I+Met-treated mice, p=0.94 relative to Metand p=0.022 relative to H-I, respectively). G Representative image of activatedmicroglia (Iba1+CD68+) at P9 (first time point of activation) in the striatum inSham mice (20x magnification). H Representative image of activated microglia (Iba1+CD68+)at P9 (first time point of activation) in the striatum in Met-treated mice (20xmagnification). I Representative image of activated microglia (Iba1+CD68+) at P9(first time point of activation) in the striatum after H-I (20x magnification).J Representative image of activated microglia (Iba1+CD68+) at P9 in the striatumafter H-I+MET (20x magnification). K Quantification of Iba1+CD68+ in the striatumat P9 across groups, expressed as percentage per unit area. There was a significantincrease of Iba1+CD68+ microglia after H-I relative to Sham (26.83 ± 1.60% Iba1+CD68+cells in Sham mice vs. 64.46 ± 9.40% in H-I-injured mice, p=0.042). This increasein Iba1+CD68+ cells was prevented by HI+Met (18.83± 8.00% Iba1+CD68+ cells in Met-treatedmice vs. 17.06 ± 9.99% Iba1+CD68+ cells in H-I+Met-treated mice, p=0.999 relativeto Met and p=0.013 relative to H-I, respectively). L Quantification of activatedmicroglia (Iba1+CD68+) in the striatum at P12 across groups, expressed as percentageper unit area. There was a significant increase of Iba1+CD68+ microglia after H-Irelative to Sham (19.58 ± 8.07% Iba1+CD68+ cells in Sham mice vs. 65.32 ± 2.32%in H-I-injured mice, p=0.0010). This increasein Iba1+CD68+ cells was prevented by HI+Met (23.93± 5.22% Iba1+CD68+ cells in Met-treatedmice vs. 23.76 ± 2.53% Iba1+CD68+ cells in H-I+Met-treated mice, p=>0.99 relativeto Met and p=0.002 relative to H-I, respectively). M Representative image of amoeboidmicroglia (Iba1+) at P9 in the SEZ in Sham mice (40x magnification). Image is representativeof the first time point of activation in the SEZ. N Representative image of amoeboidmicroglia (Iba1+) at P9 in the SEZ in Met-treated mice (40x magnification). Imageis representative of the first time point of activation in the SEZ. O Representativeimage of amoeboid microglia (Iba1+) at P9 in the SEZ in H-I mice (40x magnification).Image is representative of the first time point of activation in the SEZ. P Representativeimage of amoeboid microglia (Iba1+) at P9 in the SEZ in H-I+MET mice (40x magnification).Image is representative of the first time point of activation in the SEZ. Q Quantificationof activated microglia (Iba1+CD68+) in the SEZ at P9 across groups, expressed aspercentage per unit area. There was a significant increase of Iba1+CD68+ microgliaafter H-I relative to Sham (40.84 ± 6.53% Iba1+CD68+ cells in Sham mice vs. 86.61± 4.44% in H-I-injured mice, p=0.0060). This increase in Iba1+CD68+ cells was preventedby HI+Met (38.46± 8.60% Iba1+CD68+ cells in Met-treated mice vs. 33.60 ± 6.79% Iba1+CD68+cells in H-I+Met-treated mice, p=0.95 relative to Met and p=0.0024 relative to H-I,respectively). R Quantification of activated microglia (Iba1+CD68+) in the SEZ atP12 across groups, expressed as percentage per unit area. There was a significantincrease of Iba1+CD68+ microglia after H-I relative to Sham (38.08 ± 10.20% Iba1+CD68+cells in Sham mice vs. 85.12 ± 6.03% in H-I-injured mice, p=0.023) 85.12 ± 6.03%in H-I-injured mice, p=0.017). This increase in Iba1+CD68+ cells was prevented byHI+Met (35.53± 9.69% Iba1+CD68+ cells in Met-treated mice vs. 33.21 ± 8.81%Iba1+CD68+cells in H-I+Met-treated mice, p=0.99 relative to Met and p=0.013 relative to H-I,respectively). n=3-5 mice per group. Data presented as mean±SEM. Scale Bar: 50um. Unit area (cortex, striatum): 3x0.105 mm2.Unit area (SEZ): 3x0.045mm2. Statistics: (D, G-H, K-L) Oneway ANOVA. *p<0.050.

**Additional file 4: Figure S4.** Dose-dependentmicroglia ablation is seen following one week of daily injections of Plexxikon.A Experimental timeline following PLX 5622 administration (vehicle control,10ug/g or 50ug/g) from P8 to P15. Immunohistochemistry (IHC) or the neurosphereassay (NS) was performed on P15. B No differences in pup weight were recordedacross treatment groups. C Representative image of microglia (Iba1+, red) atP15 in the cortex in Sham mice (20x magnification). D Representative image ofmicroglia (Iba1+, red) at P15 in the cortex in mice that received 10ug PLX fromP8 to 15 (20x magnification). E Representative image of microglia (Iba1+, red)at P15 in the cortex in mice that received 50ug PLX from P8 to 15 (20xmagnification). F Quantification of the number microglia (Iba1+ cells/area) inthe cortex, reported as fold change. Microglia are significantly depleted with10ug/g PLX (1.00±0.11-fold change of Iba1+ cells in vehicle-treated mice vs.0.52±0.03-fold change Iba1+ cells in 10ug/g PLX-treated mice) (p=0.008) and50ug/g PLX (1.00±0.11-fold change of Iba1+ cells in vehicle-treated mice vs.0.18± 0.05-fold change of Iba1+ cells in 50ug/g PLX-treated mice (p=0.0004).PLX at 50ug/g results in a significantly greater loss of Iba1+ cells comparedto 10ug/g (0.52±0.03-fold change Iba1+ cells in 10ug/g PLX-treated mice vs.0.18±0.05-fold change of Iba1+ cells in 50ug/g PLX-treated mice) (p=0.033).Average number of Iba1+cells/unit area: Vehicle=67.17±7.21; 10ug/gPLX=35.28±2.29; 50ug/g PLX=12.42±3.2. G Representative image of microglia(Iba1+, red) at P15 in the striatum in Sham mice (20x magnification). HRepresentative image of microglia (Iba1+, red) at P15 in the striatum in micethat received 10ug PLX from P8 to 15 (20x magnification). I Representativeimage of microglia (Iba1+, red) at P15 in the striatum in mice that received50ug PLX from P8 to 15 (20x magnification). J Quantification of the numbermicroglia (Iba1+ cells/area) in the striatum, reported as fold change.Microglia are not depleted with PLX at 10ug/g (1.00± 0.08-fold change of Iba1+cells in vehicle-treated mice vs. 0.77±0.08-fold change Iba1+ cells in 10ug/gPLX-treated mice) (p=0.4514) but are significantly reduced with 50ug/g PLX(1.00± 0.08-fold change of Iba1+ cells in vehicle-treated mice vs.0.25±0.04-fold change of Iba1+ cells area in 50ug/g PLX-treated mice vs.vehicle (p=0.003). PLX at 50ug/g results in a significantly greater loss ofIba1+ cells compared to 10ug/g (0.77±0.08-fold change Iba1+ cells in 10ug/gPLX-treated mice vs. 0.25±0.04-fold change of Iba1+ cells in 50ug/g PLX-treatedmice) (p=0.0034). Average number of Iba1+cells/unit area: Vehicle = 32.19±2.65;10ug/g = 28.00 ± 2.65; 50ug/g PLX = 9.83±1.36. K Representative image ofmicroglia (Iba1+, red) at P15 in the SEZ in Sham mice (20x magnification). LRepresentative image of microglia (Iba1+, red) at P15 in the SEZ in mice thatreceived 10ug PLX from P8 to 15 (20x magnification). M Representative image ofmicroglia (Iba1+, red) at P15 in the SEZ in mice that received 50ug PLX from P8to 15 (20x magnification). N Quantification of the number microglia (Iba1+cells/ area) in the SEZ, reported as fold change. Microglia are significantlydepleted with 10ug/g PLX (1.00± 0.04-fold change of Iba1+ cells invehicle-treated mice vs. 0.55±0.03-fold change Iba1+ cells in 10ug/gPLX-treated mice) (p=0.0002) and 50ug/g PLX (0.31±0.01-fold change of Iba1+cells in vehicle-treated mice vs. 0.18±0.05-fold change of Iba1+ cells in50ug/g PLX-treated mice (p=0.004). PLX at 50ug/g results in a significantlygreater loss of Iba1+ cells compared to 10ug/g (0.55±0.03-fold change Iba1+cells in 10ug/g PLX-treated mice vs. 0.31±0.01fold change of Iba1+ cells in50ug/g PLX-treated mice) (p=0.0038). Average number of Iba1+cells/unit area:Vehicle=20.03±0.87; 10ug/g PLX=11.11±0.68; 50ug/g PLX=6.19±0.10±1.36. O Nodifference in neurosphere numbers were observed across treatment groups(p=0.39). Average number of neurospheres: Vehicle = 6.33±0.96; PLX: 7.33±0.42. n=3 mice per group. Data presented as mean ± SEM. Unit area (cortex, striatum):3x0.105 mm2. Unit area (SEZ): 3x0.045mm2. Statistics: (B) Two-way ANOVA; (C-E)One-way ANOVA. (O) Unpaired t-test. *p<0.050.

**Additional file 5: Figure S5.** Microglia depletion doesnot affect the numbers of astrocytes or SVZ-derived neural precursor cells. A Experimentaltimeline. PLX (50ug/g) was administered fromP8 to 15 or P8 to 22. Mice were sacrificedat P15 and P22 for IHC. B Representative image of astrocytes (GFP+, green) at P15in the cortex in Sham mice (20x magnification). C Representative image of astrocytes(GFP+, green) at P15 in the cortex in PLX-treated mice (20x magnification). D Representativeimage of astrocytes (GFP+, green) at P15 in the cortex in H-I-injured mice (20xmagnification). E Representative image of astrocytes (GFP+, green) at P15 in thecortex in H-I+PLX-treated mice (20x magnification). F Quantification of the numberof GFP+ astrocytes in the cortex at P15 across groups represented as fold change.There was no difference in the number of GFP+ astrocytes across treatment groups(1.00±0.09 GFP+ cells in Sham vs. 1.53±0.23 GFP+ cells after H-I; 1.12±0.14 GFP+cells in PLX vs. 1.46±0.14 GFP+ cells in H-I+PLX, p=0.12). Average number of GFP+cells/unitarea: Sham = 43.9±4.11; PLX = 50.62±5.09; H-I = 67.4±10.0; H-I+PLX = 64.2± 6.34.G Representative image of astrocytes (GFP+, green) at P15 in the striatum in Shammice (20x magnification). H Representative image of astrocytes (GFP+, green) atP15 in the striatum in PLX-treated mice (20x magnification). I Representative imageof astrocytes (GFP+, green) at P15 in the striatum in H-I-injured mice (20x magnification).J Representative image of astrocytes (GFP+, green) at P15 in the striatum in H-I+PLX-treatedmice (20x magnification). K Quantification of the numbers of GFP+ astrocytes inthe striatum across groups at P15 represented as fold change. There was no differencein the numbers of GFP+ astrocytes across treatment groups (1.00±0.09 GFP+ cells in Sham vs. 1.21±0.09 GFP+cells after H-I; 1.02±0.13 GFP+ cells in PLX vs. 1.25±0.04 GFP+ cells in H-I+PLX,p=0.22). Average number of GFP+cells/unit area: Sham = 32.3±3.03; PLX = 36.0±4.46;H-I = 53.2±4.98; H-I+PLX = 56.92± 8.21. L Quantification of the number of astrocytes(GFP+) in the cortex at P22 across groups, represented as fold change. No significantdifferences were found across groups at any time point examined (p=0.69). Averagenumber of GFP+cells/unit area: P8-15 Sac 22 Vehicle = 19.83±3.06; P8-15 Sac 22 PLX= 21.42±2.69; P8-22 Sac 22 Vehicle = 25.22±3.07; P8-22 Sac 22 PLX = 30.11± 3.00.M Quantification of the number of astrocytes (GFP+) in the striatum at P22 acrossgroups, represented as fold change. No significant differences were observed acrossgroups at any time point examined. (p=0.83) Average number of GFP+cells/unit area:P8-15 Sac 22 Vehicle = 24.83±2.38; P8-15 Sac 22 PLX = 24.83±2.99; P8-22 Sac 22 Vehicle= 24.89±2.61; P8-22 Sac 22 PLX = 26.33± 4.67. n= 3 mice per group. Data presentedas mean ± SEM. Scale Bar: 50um. Unit area (cortex, striatum): 3x0.105 mm2. Statistics:(A-B) One-way ANOVA. *p<0.050.

## Data Availability

The authors confirm that the data supporting the findings of this study are available within the manuscript and its supplementary materials. Raw data were generated at the University of Toronto. Derived data supporting the findings of this study are available upon request.

## Abbreviations

AMPK: AMP-Activated Protein Kinase
CD16: Cluster of Differentiation 16
CD32: Cluster of Differentiation 32
CD68: Cluster of Differentiation 68
CD206: Cluster of Differentiation 206
CSF-1: Colony Stimulating Factor 1
DMSO: Dimethyl Sulfoxide
EdU: 5-Ethynyl-2’-Deoxyuridine
GFAP: Glial Fibrillary Acidic Protein
GFP: Green Fluorescent Protein
H-I: HypoxiaIschaemia
Iba1: Ionized Calcium Binding Adaptor Molecule 1
IL-1β: Interleukin 1β
IL-6: Interleukin 6
LPS: Lipopolysaccharide
MARK2: Microtubule Affinity-Regulating Kinase 2
MCAO: Middle Cerebral Artery Occlusion
mRNA: Messenger RNA
NaCl: Sodium Chloride
NF-Kb: Nuclear Factor Kappa-b
NPC: Neural Progenitor Cell/Neural Precursor Cell
Par1b: Polarity-Regulating Kinase Partitioning Defective Pathway 1b
PBS: Phosphate-Buffered Saline
PFA: Paraformaldehyde
PLX: Plexxikon 5622
SEM: Standard Error of the Mean
SFM: Serum-Free Media
SEZ: Subependymal Zone
TLR4: Toll-like Receptor 4
TNF: Tumour Necrosis Factor
