# Supplementary figures and images for: Reduced microglia activation following metformin administration or microglia ablation is sufficient to prevent functional deficits in a mouse model of neonatal stroke
Source: J Neuroinflammation. 2022 Jun 15;19:146. doi: 10.1186/s12974-022-02487-x (PMC9199194; doi:10.1186/s12974-022-02487-x)

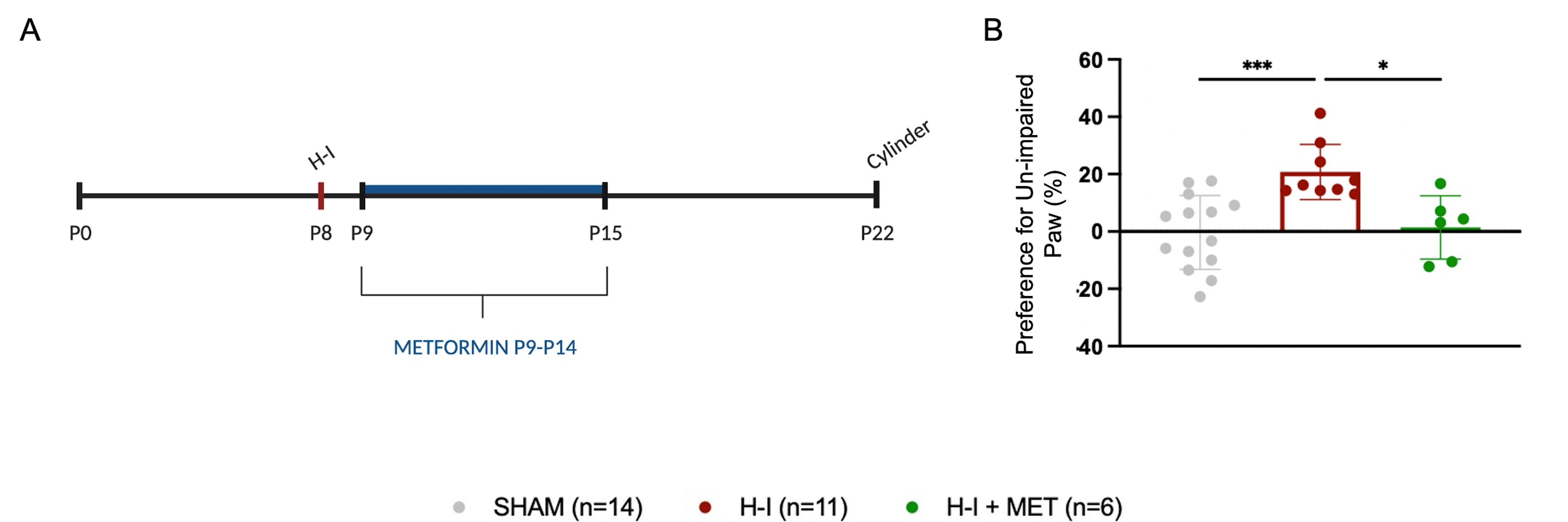

Supplement: Supplementary file 1 — Additional file 1: Figure S1. H-I-injured mice that receive metformin treatment do not display a forepaw preference on the cylinder task. A Experimental timeline. Mice receive H-I injury on P8. Metformin was administered from P9 to P15. The cylinder test is performed on P22. B Forepaw preference for the uninjured paw on the cylinder test. Sham mice do not show a paw preference. H-I injured mice are significantly impaired compared to Shams (-0.29±3.44% preference for the un-impaired paw in Sham mice vs. 20.77±3.21% in H-I-injured mice) (p=0.0004). H-I+MET are significantly improved compared to H-I only (1.42±4.50% preference for the un-impaired paw in H-I+Met mice, p=0.012) and not significantly different from Shams (p=0.95). n=6–14 mice per group. Data presented as Mean ± SEM. Statistics: (B) One-way ANOVA. *p<0.050. [file 12974_2022_2487_MOESM1_ESM.jpg]

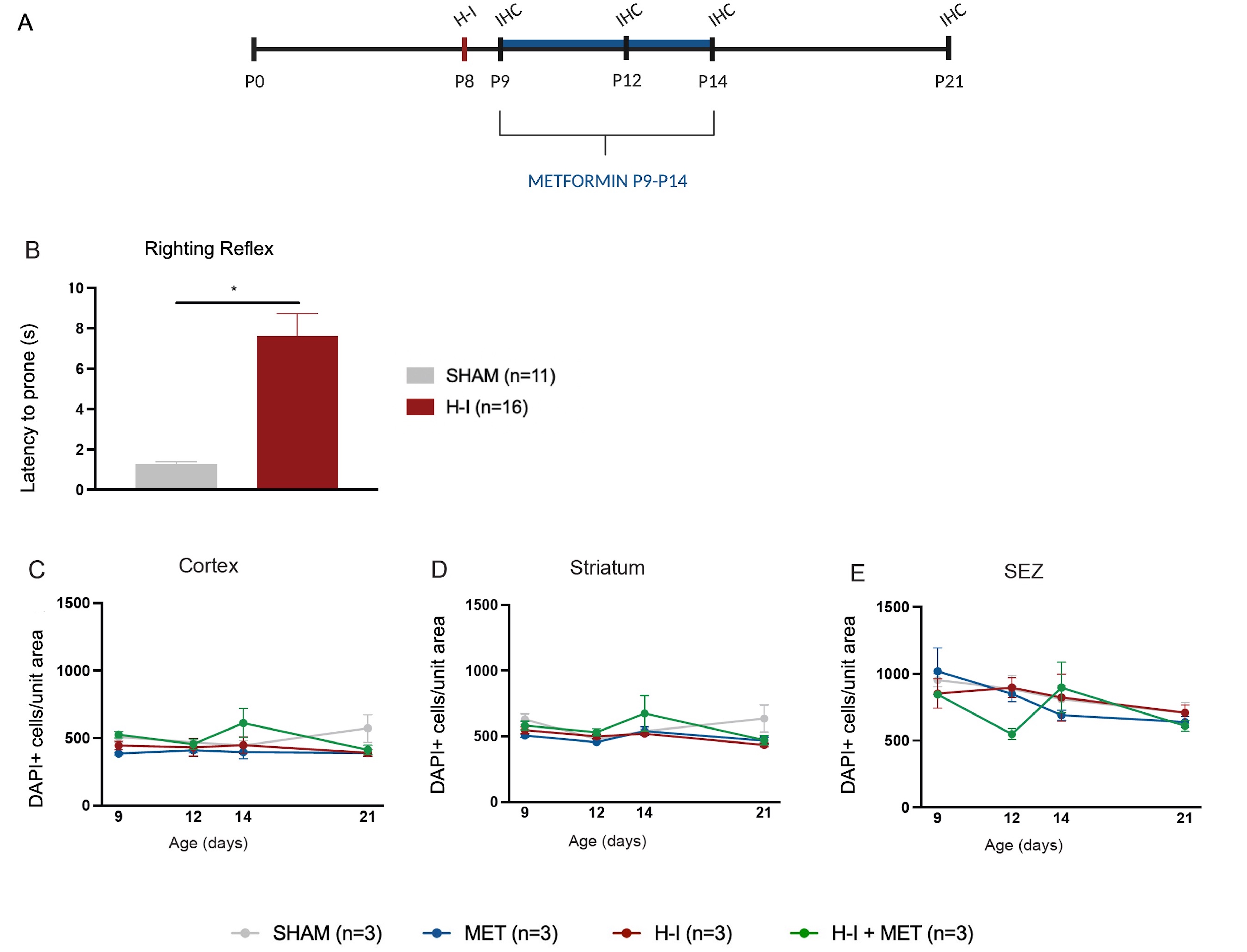

Supplement: Supplementary file 2 — Additional file 2: Figure S2. H-I injury leads to behavioural impairments but no change in cell density in the cortex, striatum and SEZ. A Experimental timeline. Mice received H-I on P8 and Metformin administration from P9 to P14. Immunohistochemistry (IHC) was performed on P9, P12, P14 and P21. Righting reflex was performed on P8 at one hour post H-I. B H-I results in increased latency to perform the righting reflex test relative to Sham (1.26±0.16s latency in Sham mice vs. 7.63 ± 1.10s in H-I-injured mice, p=7.34x10^-5^) on P8 when performed 1 hour post-injury. C H-I, Metformin and H-I+MET treatments do not affect the total number of DAPI+ cells in the cortex. D H-I, Metformin and H-I+MET treatments do not affect the total number of DAPI+ cells in the striatum. E H-I, Metformin and H-I+MET treatments do not affect the total number of DAPI+ cells in the SEZ. n=3 mice per group for immunohistochemistry. n=11–16 mice per group for behavioural testing. Data presented as mean ± SEM. Unit area (cortex, striatum): 3x0.105 mm2. Unit area (SEZ): 3x0.045mm2. Statistics: (B) Unpaired t-test; (C-E) Two-way ANOVA. *p<0.050. [file 12974_2022_2487_MOESM2_ESM.jpg]

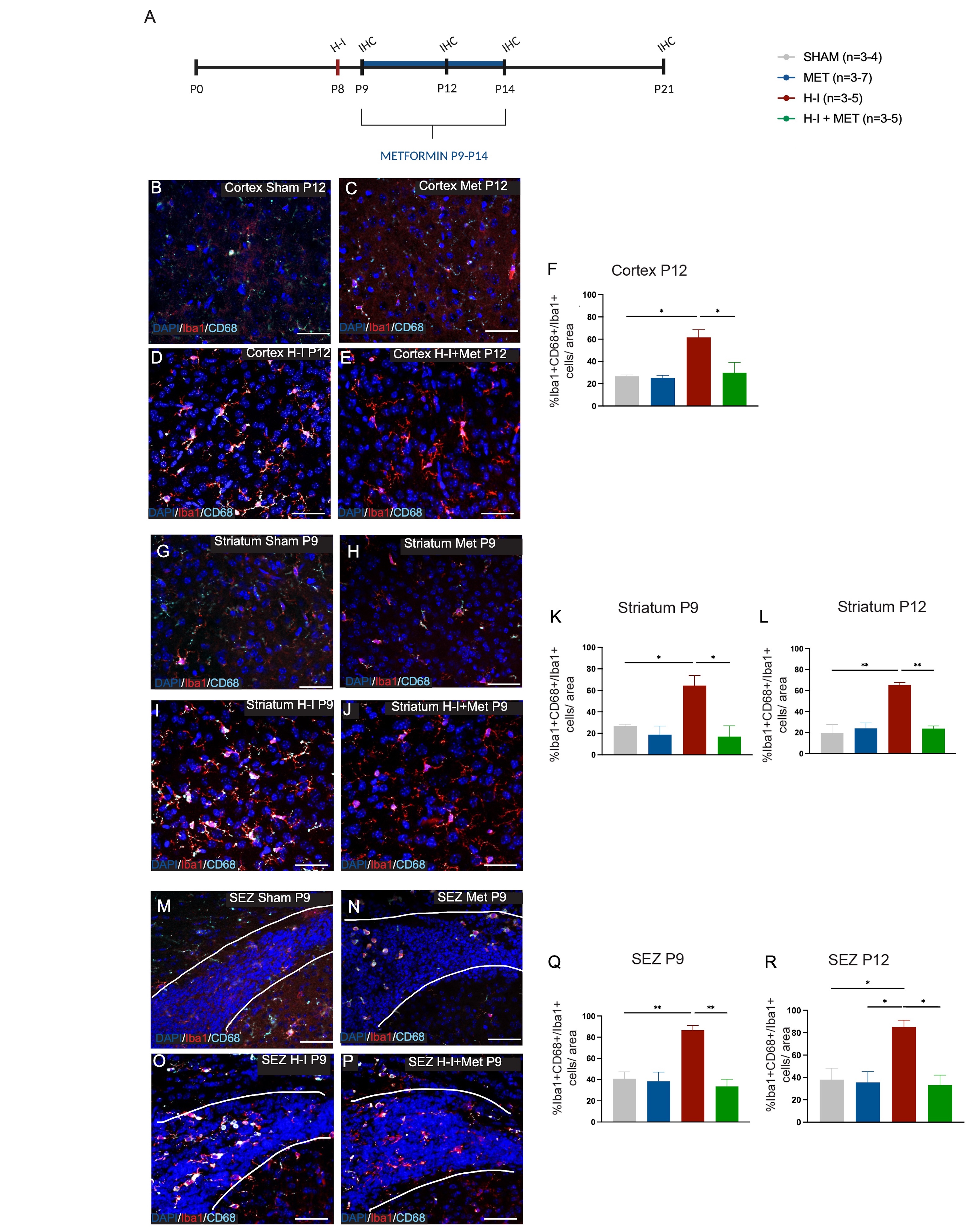

Supplement: Supplementary file 3 — Additional file 3: Figure S3. Metformin treatment reduces CD68 expression in microglia after H-I. A Experimental timeline. H-I was given onP8 and metformin administration from P9 to P14.Immunohistochemistry (IHC) was performed on P9, P12, P14 and P21. B Representativeimage of activated microglia (Iba1+ (Red) CD68+ (Cyan) double-positive cells) atP12 in the cortex in Sham mice (20x magnification). Image is representative of thefirst time point of activation. C Representative image of activated microglia (Iba1+(Red) CD68+ (Cyan) double-positive cells) at P12 in the cortex after in Met-treatedmice H-I (20x magnification). Image is representative of the first time point ofactivation. D Representative image of activated microglia (Iba1+ (Red) CD68+ (Cyan)double-positive cells) at P12 in the cortex after H-I (20x magnification). Imageis representative of the first time point of activation. E Representative imageof activated microglia (Iba1+CD68+) at P12 (first time point of activation) in thecortex after H-I+MET (20x magnification). Image is representative of the first timepoint of activation. F Quantification of Iba1+CD68+ in the cortex at P12 acrossgroups, expressed as percentage per unit area. There was a significant increaseof Iba1+CD68+ microglia after H-I relative to Sham (26.74 ± 1.24% Iba1+CD68+ cellsin Sham mice vs. 61.70 ± 6.89%in H-I-injured mice, p=0.013). This increase in Iba1+CD68+cells was prevented by HI+Met (25.22± 2.23 % Iba1+CD68+ cells in Met-treated micevs. 29.84 ± 9.30% Iba1+CD68+ cells in H-I+Met-treated mice, p=0.94 relative to Metand p=0.022 relative to H-I, respectively). G Representative image of activatedmicroglia (Iba1+CD68+) at P9 (first time point of activation) in the striatum inSham mice (20x magnification). H Representative image of activated microglia (Iba1+CD68+)at P9 (first time point of activation) in the striatum in Met-treated mice (20xmagnification). I Representative image of activated microglia (Iba1+CD68+) at P9(first time point [file 12974_2022_2487_MOESM3_ESM.jpg]

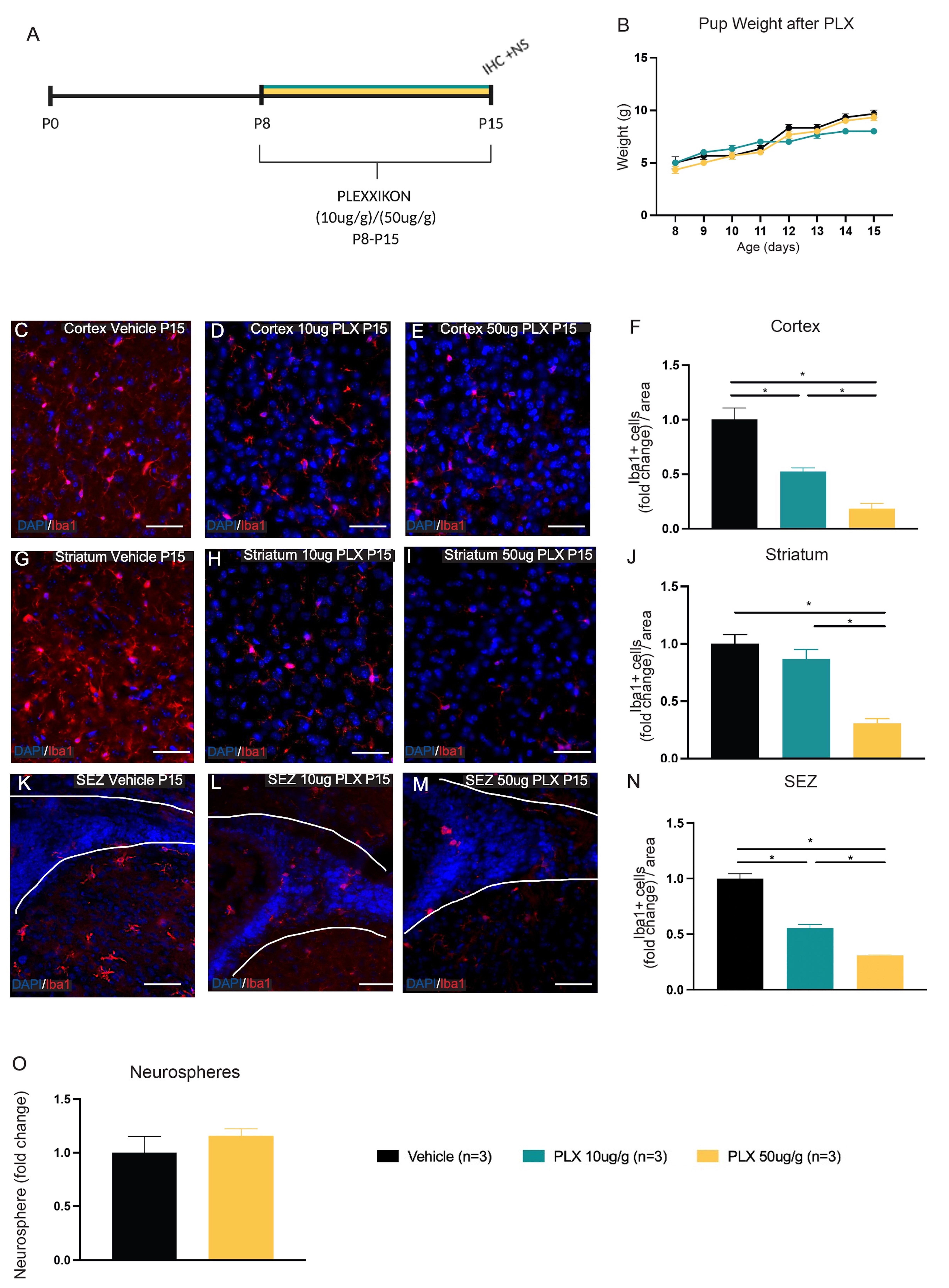

Supplement: Supplementary file 4 — Additional file 4: Figure S4. Dose-dependentmicroglia ablation is seen following one week of daily injections of Plexxikon.A Experimental timeline following PLX 5622 administration (vehicle control,10ug/g or 50ug/g) from P8 to P15. Immunohistochemistry (IHC) or the neurosphereassay (NS) was performed on P15. B No differences in pup weight were recordedacross treatment groups. C Representative image of microglia (Iba1+, red) atP15 in the cortex in Sham mice (20x magnification). D Representative image ofmicroglia (Iba1+, red) at P15 in the cortex in mice that received 10ug PLX fromP8 to 15 (20x magnification). E Representative image of microglia (Iba1+, red)at P15 in the cortex in mice that received 50ug PLX from P8 to 15 (20xmagnification). F Quantification of the number microglia (Iba1+ cells/area) inthe cortex, reported as fold change. Microglia are significantly depleted with10ug/g PLX (1.00±0.11-fold change of Iba1+ cells in vehicle-treated mice vs.0.52±0.03-fold change Iba1+ cells in 10ug/g PLX-treated mice) (p=0.008) and50ug/g PLX (1.00±0.11-fold change of Iba1+ cells in vehicle-treated mice vs.0.18± 0.05-fold change of Iba1+ cells in 50ug/g PLX-treated mice (p=0.0004).PLX at 50ug/g results in a significantly greater loss of Iba1+ cells comparedto 10ug/g (0.52±0.03-fold change Iba1+ cells in 10ug/g PLX-treated mice vs.0.18±0.05-fold change of Iba1+ cells in 50ug/g PLX-treated mice) (p=0.033).Average number of Iba1+cells/unit area: Vehicle=67.17±7.21; 10ug/gPLX=35.28±2.29; 50ug/g PLX=12.42±3.2. G Representative image of microglia(Iba1+, red) at P15 in the striatum in Sham mice (20x magnification). HRepresentative image of microglia (Iba1+, red) at P15 in the striatum in micethat received 10ug PLX from P8 to 15 (20x magnification). I Representativeimage of microglia (Iba1+, red) at P15 in the striatum in mice that received50ug PLX from P8 to 15 (20x magnification). J Quantification of the numbermicroglia (Iba1+ cells/area) in the striatum, reported as fold change [file 12974_2022_2487_MOESM4_ESM.jpg]

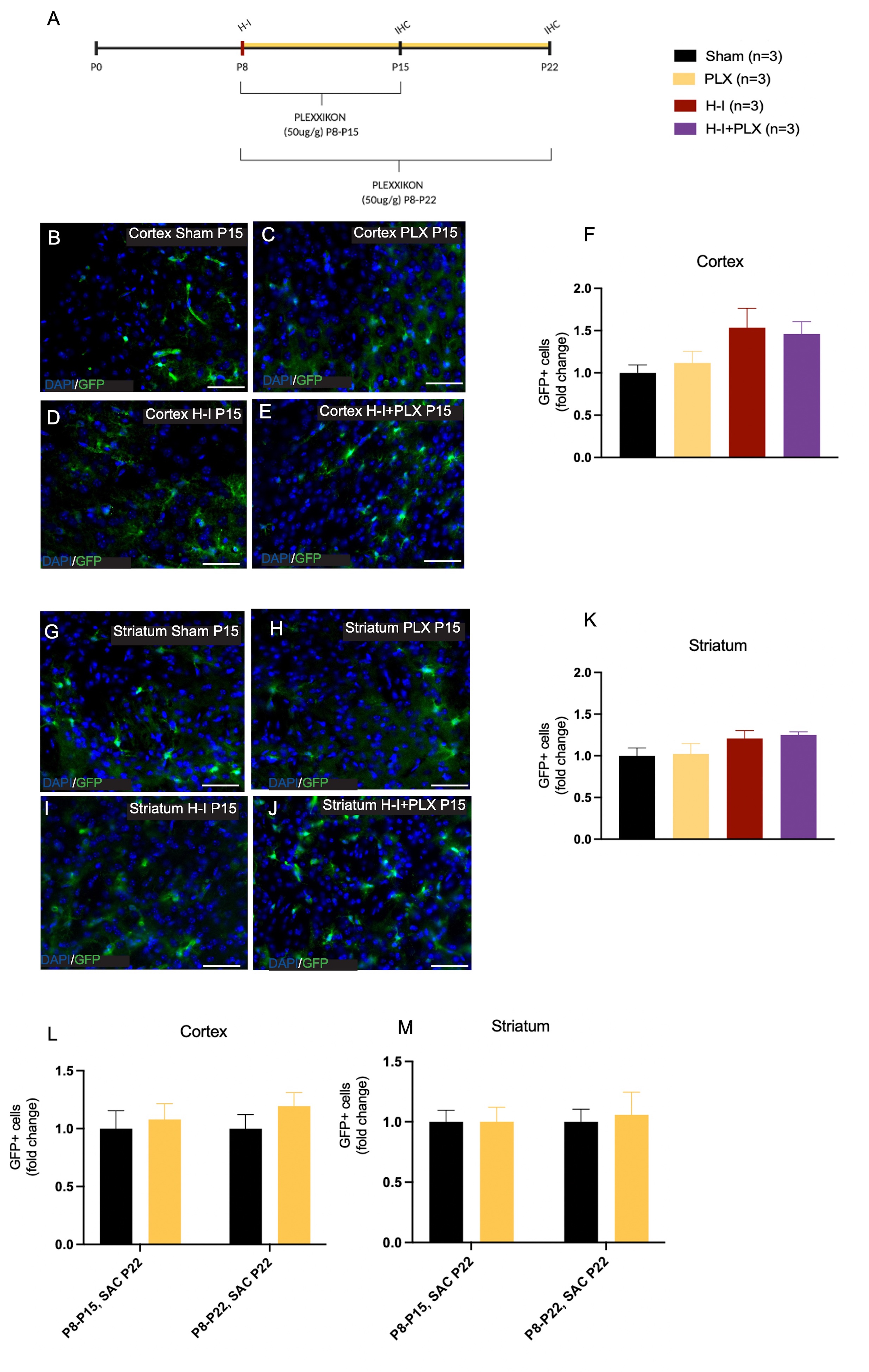

Supplement: Supplementary file 5 — Additional file 5: Figure S5. Microglia depletion doesnot affect the numbers of astrocytes or SVZ-derived neural precursor cells. A Experimentaltimeline. PLX (50ug/g) was administered fromP8 to 15 or P8 to 22. Mice were sacrificedat P15 and P22 for IHC. B Representative image of astrocytes (GFP+, green) at P15in the cortex in Sham mice (20x magnification). C Representative image of astrocytes(GFP+, green) at P15 in the cortex in PLX-treated mice (20x magnification). D Representativeimage of astrocytes (GFP+, green) at P15 in the cortex in H-I-injured mice (20xmagnification). E Representative image of astrocytes (GFP+, green) at P15 in thecortex in H-I+PLX-treated mice (20x magnification). F Quantification of the numberof GFP+ astrocytes in the cortex at P15 across groups represented as fold change.There was no difference in the number of GFP+ astrocytes across treatment groups(1.00±0.09 GFP+ cells in Sham vs. 1.53±0.23 GFP+ cells after H-I; 1.12±0.14 GFP+cells in PLX vs. 1.46±0.14 GFP+ cells in H-I+PLX, p=0.12). Average number of GFP+cells/unitarea: Sham = 43.9±4.11; PLX = 50.62±5.09; H-I = 67.4±10.0; H-I+PLX = 64.2± 6.34.G Representative image of astrocytes (GFP+, green) at P15 in the striatum in Shammice (20x magnification). H Representative image of astrocytes (GFP+, green) atP15 in the striatum in PLX-treated mice (20x magnification). I Representative imageof astrocytes (GFP+, green) at P15 in the striatum in H-I-injured mice (20x magnification).J Representative image of astrocytes (GFP+, green) at P15 in the striatum in H-I+PLX-treatedmice (20x magnification). K Quantification of the numbers of GFP+ astrocytes inthe striatum across groups at P15 represented as fold change. There was no differencein the numbers of GFP+ astrocytes across treatment groups (1.00±0.09 GFP+ cells in Sham vs. 1.21±0.09 GFP+cells after H-I; 1.02±0.13 GFP+ cells in PLX vs. 1.25±0.04 GFP+ cells in H-I+PLX,p=0.22). Average number of GFP+cells/unit area: Sham = 32.3±3.03; PLX = 36.0±4.46;H [file 12974_2022_2487_MOESM5_ESM.jpg]
